# Halving premature death and improving quality of life at all ages: cross-country analyses of past trends and future directions

**DOI:** 10.1016/S0140-6736(24)02417-6

**Published:** 2024-12-14

**Authors:** Ole F Norheim, Angela Y Chang, Sarah Bolongaita, Mariana Barraza-Lloréns, Ayodamope Fawole, Lia Tadesse Gebremedhin, Eduardo González-Pier, Prabhat Jha, Emily K Johnson, Omar Karlsson, Mizan Kiros, Sarah Lewington, Wenhui Mao, Osondu Ogbuoji, Muhammad Pate, Jennifer L Sargent, Xuyang Tang, David Watkins, Gavin Yamey, Dean T Jamison, Richard Peto

**Affiliations:** a Bergen Centre for Ethics and Priority Setting, Department of Global Public Health and Primary Care, University of Bergen, Bergen, Norway; b Danish Centre for Health Economics and Department of Public Health, Danish Institute for Advanced Study, University of Southern Denmark, Odense, Denmark; c Blutitude, Mexico City, Mexico; d Duke Global Health Institute, Duke University, Durham, NC, USA; e Harvard Ministerial Leadership Programme, Division of Policy Translation and Leadership Development, Harvard TH Chan School of Public Health, Harvard University, Boston, MA, USA; f Palladium Group, Washington, DC, USA; g Center for Global Health Research, Dalla Lana School of Public Health, University of Toronto, Toronto, Canada; h Nuffield Department of Population Health, University of Oxford, Oxford, UK; i Health Data Research, University of Oxford, Oxford, UK; j Minister of Health and Social Welfare, Ministry of Health, Abuja, Nigeria; k Department of Global Health, University of Washington, Seattle, WA, USA; l Department of Epidemiology and Biostatistics, University of California, San Francisco, CA, USA

## Abstract

**Background:**

Although death in old age is unavoidable, premature death—defined here as death before age 70 years—is not. To assess whether halving premature mortality by 2050 is feasible, we examined the large variation in premature death rates before age 70 years and trends over the past 50 years (1970–2019), covering ten world regions and the 30 most-populous nations. This analysis was undertaken in conjunction with the third report of *The Lancet Commission on Investing in Health: Global Health 2050: the path to halving premature death by mid-century*.

**Methods:**

In this cross-country analysis of past mortality trends and future directions, all analyses on the probability of premature death (PPD) were conducted using life tables from the UN World Population Prospects 2024. For each sex, country, and year, probability of death was calculated from these life tables with 1-year age-specific mortality rates.

**Findings:**

Globally, PPD decreased from 56% in 1970 to 31% in 2019, although some countries saw reversals because of conflict, social instability, or HIV and AIDS. Child mortality has decreased faster than adult mortality. Among all countries, 34 halved their PPD over three decades between 1970 and 2019. Among the 30 most-populous countries, seven countries, with varying levels of baseline PPD and income, halved their PPD in the past half century. Seven of the most-populous countries had average annual rates of improvement in the period 2010–19 that, if sustained, could lead to a halving of PPD by 2050, including Korea (3·9%), Bangladesh (2·8%), Russia (2·7%), Ethiopia (2·4%), Iran (2·4%), South Africa (2·4%), and Türkiye (2·3%).

**Interpretation:**

Halving premature death by 2050 is feasible, although substantial investments in child and adult health are needed to sustain or accelerate the rate of improvement for high-performing and medium-performing countries. Particular attention must be paid to countries with very low or a worsening rate of improvement in PPD. By reducing premature mortality, more people will live longer and more healthy lives. However, as people live longer, the absolute number of years lived with chronic disease will increase and investments in services reducing chronic disease morbidity are needed.

**Funding:**

The Norwegian Agency for Development Cooperation, the Bill & Melinda Gates Foundation, and a Norwegian Research Council Centre of Excellence grant.

## Introduction

For most people in the world, living to age 70 years is a reasonable expectation, and a realistic indicator of progress towards the mid-21st century would be probability of premature death (death before age 70 years). Historically, under-five mortality (probability of death before age 5 years) has been a widely used and is a well understood indicator of progress in global health. As child mortality and fertility decline and a demographic shift to older-aged populations occurs, non-communicable diseases (NCDs) have become more prominent in most countries.^1^ Thus, in many regions of the world, avoidable adult deaths is now a major concern. We chose age 70 years as the cutoff value for two reasons: first, because global life expectancy at birth is now around age 70 years. Saving lives in people younger than this life-expectancy age will increase both life expectancy and lifespan equality;^2^ and second, because the upper age limit for the sustainable development goal NCD mortality target 3.4 is 70 years. If halving premature death in all countries could be achieved, the absolute gap in probability of premature death between low-income and high-income countries would be reduced.

A 2014 review of national mortality trends to help quantify the United Nations Sustainable Development Goal for health^3^, ^4^ concluded that a 40% reduction in premature deaths, relative to those that would have resulted from a continuation of 2010 death rates, could be achieved by 2030, or soon afterward at least in areas free of war, other major effects of political disruption, or a major new epidemic.^4^ A decade later, the world has been through a major pandemic and continues to experience political disruption, nationalism, inflation, climate change, and violent conflict in Africa, the Middle East, Europe, and elsewhere.^5^ Against this background, we used the latest-available mortality estimates to explore mortality and mortality trends and determined whether the substantial gains in health improvement and reductions in premature death observed historically, before the COVID-19 pandemic, can be sustained.

**Research in context**

**Evidence before this study**

Global trends in age-specific mortality, life expectancy and burden of disease are regularly published. Only one previous study has studied trends in premature death defined as the probability of death between birth and age 70. We updated this study beyond year 2010, analysed trends in premature death and explored whether halving premature death and improving quality of life at all ages by 2050 is feasible for each country in the world.

**Added value of this study**

Our study showed that, historically, a large number of countries were able to halve premature death in three decades or less, and that recent rates of improvements indicate that several countries are on track to halve or substantially reduce the probability of premature death by 2050. Countries with improved premature mortality also achieve a higher healthy life expectancy.

**Implications of all the available evidence**

The available evidence indicates that halving premature death and improving quality of life at all ages by 2050 is feasible. Looking ahead, countries not on track can learn from best-performing countries and achieve the same rate of improvement as their better-performing regional neighbours through benchmarking, sustained and substantial investments, and addressing the major causes of premature death.

We aimed to document mortality trends in the past half century and explore whether halving premature death by 2050 compared to pre-pandemic levels (2019) is feasible for each country in the world. To assess feasibility, we explored whether one or more countries historically have achieved a halving of premature death in about three decades or less. In addition, we estimated which countries had the necessary 2010–19 rate of change in probability of premature death (PPD) that, if continued, would lead to a halving of PPD over the next three decades. We also explored the rate of improvement in PPD for the 30 most-populous countries (in our baseline year 2019) and the ten global regions as defined by the third *Lancet Commission on Investing in Health* (CIH3).^5^ For selected countries, we provide an in-depth discussion of past trends and prospects for the future. These countries were chosen to reflect both good and less well performing populous countries.

Our analysis was a crucial input into the recently published CIH3 report.^5^ In the CIH3 report, discussion of the feasibility of achieving a 50% reduction in PPD by 2050 is followed by analysis of how countries that choose to do so can drive down their PPD and their burden of morbidity. In particular, the report emphasises the importance of focusing on 15 high-priority conditions—eight related to infectious disease and maternal conditions and seven related to non-communicable diseases and injuries—and on scaling up financing to develop and deliver new health technologies.

## Methods

### Probability of premature death

In this cross-country analysis of past mortality trends and future directions, we defined premature death as death before age 70 years. PPD is defined as the probability that a child born in the indicated year would die before age 70 years if the age-specific death rates prevailing at the year of birth were to continue unchanged (_70_q_0_). PPD provides a snapshot of mortality probability in the indicated year and is thus independent of the age distribution of the population. PPD differs conceptually from indicators involving actual numbers of deaths that mix death rates with the age distribution of the population.^4^

We used PPD as an indicator to measure trends in health improvement since 1970, that is to say during the past half century. All analyses on PPD were done using life tables from the UN World Population Prospects 2024 (WPP 2024).^6^ For each sex, country, and year, probability of death was calculated from these life tables with 1-year age-specific mortality rates as,

$${\;}_{\text{n}}{\text{q}}_{\text{x}}=\frac{1_{\text{x}}-1_{\text{x}+\text{n}}}{1_{\text{x}}}$$

where l_x_ and l_x + n_ are the number of people starting in the cohort at age x and at age x + n. For example, PPD (_70_q_0_) is the number of people dying before age 70 years over the starting population at age 0, or,

$$\text{PPD}={\;}_{70}{\text{q}}_{0}=\frac{1_{0}-1_{70}}{1_{0}}$$

is the probability of dying between age 0 years and exact age 70 years, l_0_ is the number of people alive at age 0 years (100 000 in standard life tables), and l_70_ is the number of people alive at exact age 70 years (ie, those who did not die before their 70th birthday). For this Article, we use annual rate of improvement to indicate the decline in PPD. A faster rate of improvement is desired. A few countries have a worsening or increasing rate of change, which is indicated with a plus sign in all tables. We also calculated the probabilities of dying between ages 0–14 years (_15_q_0_), 15–49 years (_35_q_15_), and 50–69 years (_20_q_50_) using the same equation. For example, for the age group 50–69 years, _20_q_50_ is the probability of death between age 50 years and exact age 70 years, conditional on survival to exact age 50 years.

### Trends in the probability of premature death

To explore whether halving the probability of premature death by 2050 is feasible, we looked at variations in the rates of change in previous decades for the world's major regions (central and eastern Europe, Latin America and the Caribbean, the Middle East and north Africa, the north Atlantic [western Europe and Canada], sub-Saharan Africa, central Asia, and the western Pacific and southeast Asia) and the world's 30 most-populous countries. We then assessed which countries, through improved and targeted health policies, could or could not realistically achieve a halving of premature death. To calculate a 50% reduction in premature death, we chose the year 2019 as our baseline. PPD levesl for the period 2020–23 were substantially affected by the COVID-19 pandemic and are therefore inappropriate as baseline years for assessing progress. To determine feasibility of what CIH3 calls 50 by 50, a 50% reduction in the probability of premature death by 2050, we estimated changes in the probability of premature death from the past half century, and calculated the average annual rate of improvement in PPD (AARI) with the following equation,

$$\text{AARI}={\left(\frac{{\text{q}}_{\text{t}}}{{\text{q}}_{\text{t}+\text{n}}}\right)}^{\frac{\text{1}}{\text{n}}}-1$$

where q_t_ is the probability of premature death at time t and q_t + n_ is the probability after a further n years. The rate of improvement needed to reach a 50% reduction was estimated by setting the probability in year 2050 (q_2050_) at half the level of the probability in 2019 (q_2019_). The required rate of improvement to reach a halving of PPD in 31 years (ie, by 2050 from a 2019 baseline) is the same for all regions and countries, 2·2%. We also calculated the rate of improvement for each aforementioned age group.

The required rate of improvement depends only on the level of PPD in our baseline year and the set target and does not depend upon past rate of improvement. We then compared the observed rate of improvement over the past decade (2010–19) with the required rate of improvement over the next three decades. On the basis of the annual rate of improvement in the period 2010–19, we pragmatically grouped countries into three categories: high (>2·2%), medium (2·2–1·0%), and low (<1·0%) rate of PPD change. The method for decomposition of changes in PPD by age is provided in the appendix.

### Regions

Since we explore historical health trends in the past half century and discuss feasibility of continued or improved rate of improvement for the next 30 years, World Bank regional classifications of countries by income are not appropriate. For example, more than 30 countries have transitioned from low-income status, defined by the World Bank classification, to middle-income status since 2001.^7^ In addition, the income classification cutoffs have changed. We therefore follow CIH3 and classify countries in geographical regions with somewhat similar characteristics in terms of disease burden and economic development (appendix p 6). The world's three most populous countries (India, China, and the USA) are separated out as regions given that they would dominate all trends if included in other regions. The north Atlantic region includes western Europe and Canada.

### Role of the funding source

The funders of the study had no role in study design, data collection, data analysis, decision to publish, or writing of this report.

## Results

**Figure 1:**
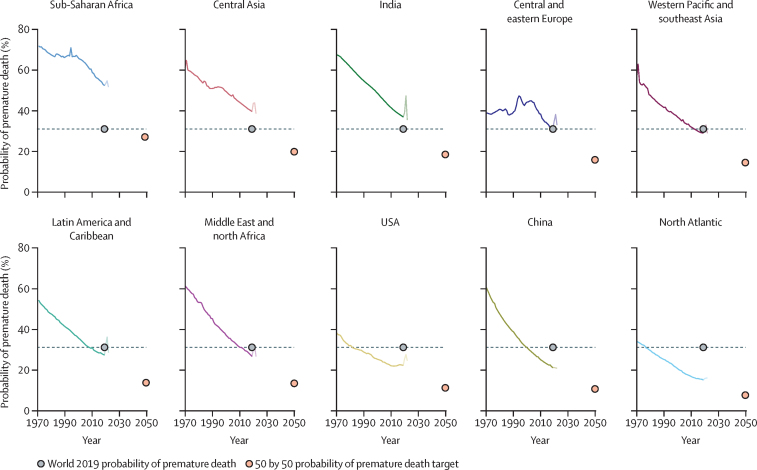
Progress in probability of PPD by CIH3 region for both sexes, 1970–2023 The horizontal dashed line and black dot show PPD for the world in 2019. The red dot indicates halved region-specific PPD in year 2050 compared to 2019 (the baseline year). The north Atlantic region includes western Europe and Canada. For China, the graph shows data for 1970–2020. CIH3=third commission on investing in health. PPD=probability of premature death.

There have been major improvements in PPD over the past half century, but disparities across regions, countries, and sex remain in the levels of and trends in PPD. In 2019, before the COVID-19 pandemic, PPD ranged from 52% in sub-Saharan Africa to 15% in the north Atlantic (table 1). The PPD in sub-Saharan Africa, central Asia, and India was higher than the world average (31%; figure 1).

**Table 1:** PPD in 2019 and average annualised rate of improvement in 2010–19 in CIH3 regions and for the world, for both sexes combined

	**PPD**	**Rate of improvement**
Sub-Saharan Africa	52%	1·2%
Central Asia	40%	1·2%
India	37%	1·3%
Central and eastern Europe	32%	2·1%
Western Pacific and southeast Asia	29%	1·1%
Latin America and Caribbean	27%	1·4%
Middle East and north Africa	26%	1·7%
USA	22%	+0·1%
China	21%	1·8%
North Atlantic (western Europe and Canada)	15%	1·2%
World	31%	1·3%

Regions are ranked by PPD. CIH3=third commission on investing in health. PPD=probability of premature death.

Central and eastern Europe showed little improvement over the past century, and even saw an increase in PPD between 1970 and 2005, but had the greatest improvement from 2010 to 2019 (2·1%). All other regions showed substantial improvements in PPD from 1970 to 2019, and five regions had equal or higher rates of improvement than the world average (1·3%; table 1). China showed a strong rate of improvement in PPD (1·8%), the improvement in India was similar to the global mean (1·3%), whereas the USA was the only region to observe a (small) worsening trend over the past decade (+0·1%).

**Figure 2:**
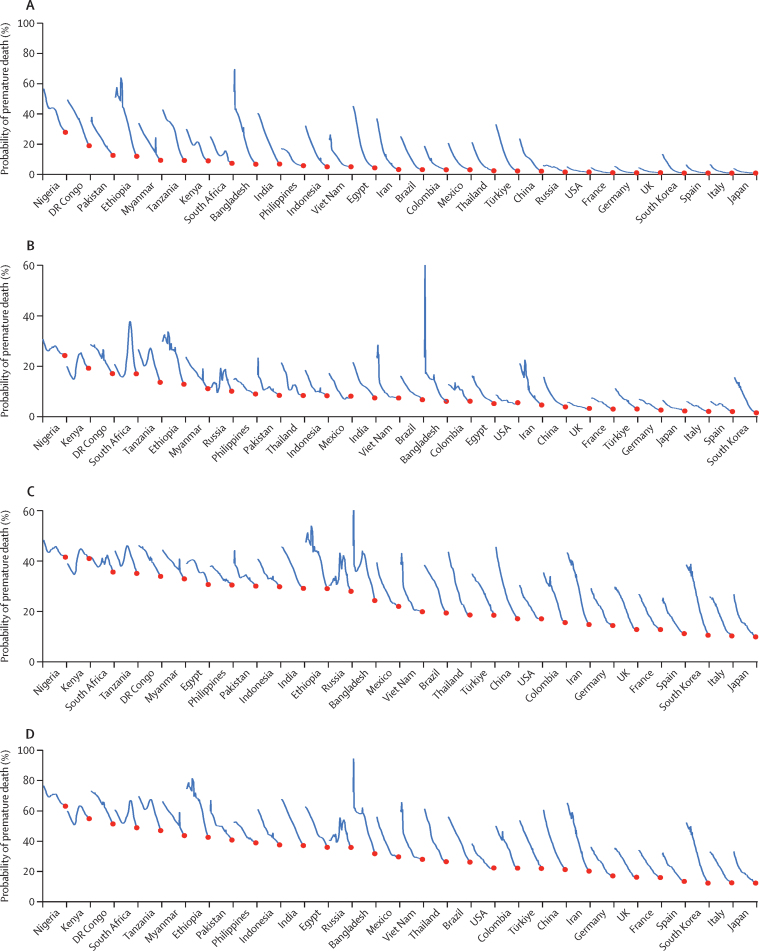
Age-specific mortality trends for both sexes in 1970–2019, for the 30 most populous countries Probability of a live-born infant dying between ages 0 years and 14 years (A). Probability of dying between ages 15 years and 49 years, conditional on being alive at 15 years (B). Probability of dying between ages 50 years and 69 years, conditional on being alive at 50 years (C). Probability of premature death (ie, dying between ages 0 years and 69 years (D). The probability of dying over a particular age range in one particular calendar year is determined by the average of the separate age-specific mortality rates within that age range in that 1 year. Hence, a sudden but transient mortality shock caused by a war, natural disaster, or epidemic produces a transient high value that shows what would happen if, purely hypothetically, the age-specific mortality rates in that one calendar year were to persist indefinitely.^6^ Countries are ranked by probability of premature death (PPD) at the mortality rates of 2019.

Levels of and trends in PPD also varied within regions. We calculated the trends in overall PPD for the 30 most-populous countries in the world and for age groups of 0–14 years, 15–49 years, and 50–69 years (figure 2).

For all age groups, substantial improvements in PPD were observed, with variations across countries. All countries saw improved PPD at ages 0–14 years (with a few periods of worsening PPD for Nigeria, Kenya, and Russia), at 15–49 years except in Kenya and South Africa because of the burden of HIV and AIDS (with a few periods of worsening PPD for several other countries caused by war and political turmoil), at 50–69 years except in Kenya, and in all countries at ages 0–69 years. Large increases in PPD were seen for some subgroups in countries substantially affected by HIV and AIDS (southern and eastern Africa around 1990), alcohol overconsumption (Russia around 2000), or war and famine (Bangladesh around 1971 and Ethiopia in the 1980s). These trends are examined in detail for selected countries in the Discussion.

From 2010 to 2019, the global annual rate of improvement in PPD was 1·3% for both sexes combined (table 1). Of the 30 most populous countries, seven had a rate of improvement equal to or better than 2·2% (the required rate to achieve 50 by 50) from 2010 to 2019 (table 2). Conversely, nine of 30 countries had a rate of improvement lower than 1·0%, meaning the implied improvement would be less than a third.

**Table 2:** Level of PPD in 2019, average annual rate of improvement in 2010–19, and implied rate of change by 2050 (if this rate of improvement is sustained) in the 30 most populous countries

	**PPD in 2019**	**Rate of improvement in 2010–19**	**Implied reduction in PPD in 2050, compared with 2019**
**Countries with rates of improvement higher than 2·2%**			
South Korea	12%	3·9%	71%
Bangladesh	32%	2·8%	58%
Russia	36%	2·7%	57%
Ethiopia	42%	2·4%	52%
Iran	20%	2·4%	52%
South Africa	49%	2·4%	53%
Türkiye	22%	2·3%	52%
**Countries with rates of improvement between 1**·**0% and 2**·**2%**			
China	21%	1·9%	44%
Japan	12%	1·9%	44%
Tanzania	47%	1·9%	44%
Brazil	26%	1·6%	40%
Egypt	36%	1·6%	40%
Colombia	22%	1·5%	38%
Italy	12%	1·5%	37%
Spain	13%	1·4%	35%
India	37%	1·3%	33%
France	16%	1·2%	32%
Myanmar	44%	1·2%	31%
DR Congo	51%	1·1%	29%
Indonesia	37%	1·0%	27%
UK	16%	1·0%	26%
**Countries with rates of improvement lower than 1**·**0%**			
Germany	17%	0·9%	23%
Pakistan	41%	0·9%	25%
Thailand	26%	0·8%	21%
Kenya	55%	0·6%	17%
Philippines	39%	0·5%	16%
Mexico	29%	0·4%	13%
Viet Nam	28%	0·4%	10%
Nigeria	63%	0·3%	9%
USA	22%	+0·1%	+3%
**World**	**31%**	**1·3%**	**33%**

Ranked according to rate of improvement in 2010–19.

Among all countries in the world, those with minimal decline or increasing PPD are highly unlikely to achieve a halving of PPD by 2050. Some of these countries, Libya, Yemen, Syria, Jamaica, Mexico, and Venezuela, are marked by war, violent political conflict, or political disruption, whereas for others (the USA, Costa Rica, and Cuba), the minimal decline or increase in PPD is harder to explain (appendix pp 15–16).

We also examined whether any country had historically achieved a halving of PPD in three decades or less. Among the 30 most populous countries, seven countries halved their PPD over 31 years or less in the past half century (the required and fastest time to achieve halving between 2019 and 2050 is given in parentheses): Bangladesh (1991–2022), Iran (1983–2006), China (1970–2001), Viet Nam (1972–1995), South Korea (1992–2011), Italy (1983–2012), and Japan (1970–2001). When looking at countries beyond the most populous, we found that among all countries in the world, 34 countries in total halved their PPD (appendix pp 15–16) over 31 years or less between 1970 and 2019. These historical experiences from diverse countries showed that halving PPD over three decades is feasible. Halving occurred in countries starting with both high initial PPD (eg, Algeria and Viet Nam) and low initial PPD (eg, Italy and Norway), and across all income levels.

There was no statistically significant correlation between initial PPD and rates of improvement in the period of 2010 to 2019 (appendix p 10). For example, South Korea had the highest rate of improvement but with low initial PPD, whereas Ethiopia also had high rate of improvement but from a higher initial PPD (table 2).

**Figure 3:**
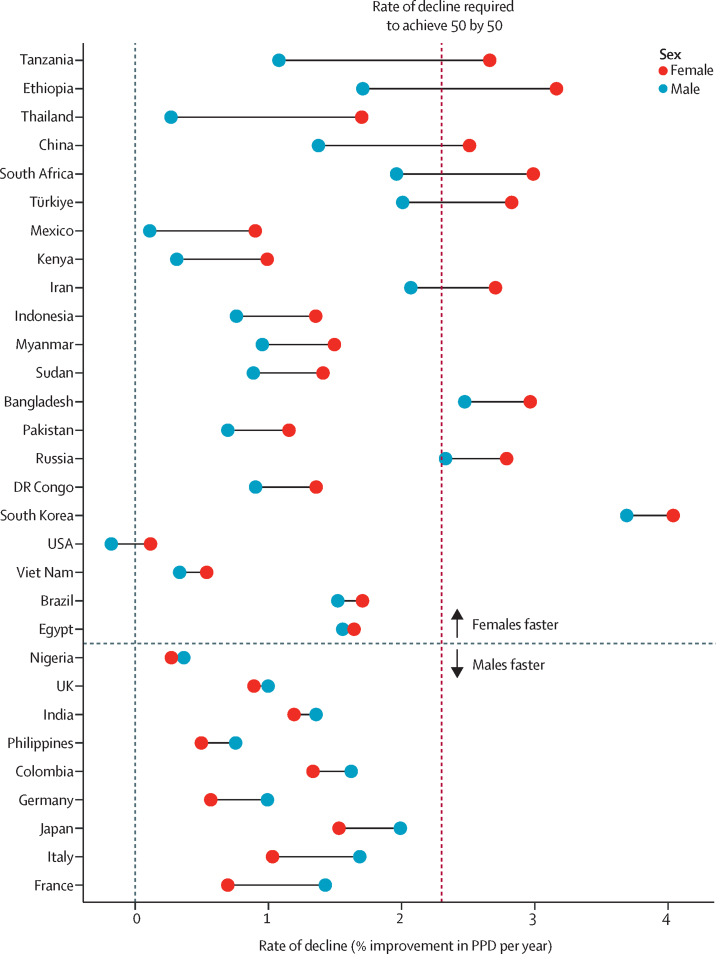
Rate of decline in PPD in the period 2010–19 by sex, for the 30 most populous countries

In all countries, females had lower PPD than males in 2019. The global rate of improvement in PPD by sex were generally higher for females than for males. In the period 2010–19, the level of PPD decreased by 1·5% annually for females and 1·1% for males (appendix p 18). In the most populous countries, rates of improvements were more favourable for females in 21 of 30 countries (figure 3; for all disaggregated results by sex, see appendix pp 18–24). However, the pattern was not uniform. For example, the gap in PPD between females and males narrowed in the USA between 1970 and 2010, whereas in Thailand, the gap increased between 1985 and 2019 (appendix p 11).

For the world, changes in PPD since 1970 have largely been driven by improvements at ages 50–69 years (appendix p 12). In the period 2010–19, about 50% of the improvements in PPD were caused by improvements in this age group, followed by the age groups 0–14 years and 15–49 years (about 23%). In the north Atlantic, the proportion contributing to the decrease in PPD has been about 70% from ages 50–69 years since the 1970s. In sub-Saharan Africa, the age group of 50–69 years contributed 40% to changes in PPD in the period 2010–19. This contribution is largely because most deaths increasingly occur in older age groups.

## Discussion

Substantial improvements in premature mortality in the past half century have been achieved, but there are also disparities across regions, countries, and sexes in levels and trends. Nevertheless, we found that 34 diverse countries halved their PPD in three decades or less and seven of the 30 most populous countries have an implied rate of improvement towards 2050 that, if sustained, could lead to a halving of PPD. The absolute gap between countries with high and low levels of PPD would also be reduced.

One striking finding is the higher rate of improvement in PPD for females over males in most countries. The reasons for this disparity warrant further scrutiny. Disparities in PPD within countries across geographical regions, income, and level of education should also be studied.

China has achieved good progress in population health over the past half century, reducing its PPD from 61% in 1970 to 21% in 2019. There have been substantial reductions in the probabilities of dying between ages 0–14 years, 15–49 years, and 50–69 years during this period. The rate of improvement in PPD has consistently remained at about 2% or more, with the most considerable progress occurring at 2·6% in the 1970s, followed by 2·3% in the 2000s. China's initial achievement in reducing premature death is largely because of its success in reducing maternal and infant mortality.^8^ Rapid economic growth, poverty alleviation efforts, and universal education programmes have also contributed to health advancements. Improving access to care and enhancing financial protection and population health were also important—these improvements were achieved through the Government's commitment to universal access to basic health care, increasing public funding for health from 1% to more than 3% of gross domestic product (GDP) to fund a universal health-insurance programme, and implementation of free national essential public health programmes (including HIV and AIDS and tuberculosis).^9^ Rural health insurance rolled out from 2003–08 might have saved about one million lives per year.^10^ However, inequality in quality of care by geographical and socioeconomic status presents another challenge.^11^, ^12^

Ethiopia made substantial progress in reducing PPD between 1991 and 2019, and since 2000, its rate of improvement has been among the fastest in sub-Saharan Africa.^13^ The largest contributions to PPD decline are from reductions in maternal and child health conditions and communicable diseases such as HIV and AIDS, tuberculosis, and malaria.^14^ These can be attributed to reforms within and outside the health sector, prioritising rural communities and primary health care that resulted in the decentralisation of health service delivery, community empowerment, and better access to primary health care. Between 1990 and 2019, GDP per capita increased from US$110 to $840,^15^ the proportion of people living in poverty was halved from 48% to 24%,^16^ literacy doubled from 27% to 52%,^17^ access to basic drinking water tripled from 13% to 38%,^18^ and total fertility decreased from 7·2 to 4·3 children per woman.^19^ The country also enjoyed peace and security between 2000 and 2020. The ongoing civil conflicts in Ethiopia since 2021 and the global crisis have hindered economic growth and could have long-term population-health effects on the country.

From 1970 to 2019, Nigeria showed a 13% decrease in PPD, with most progress occurring between 1970 and 1975 and between 2000 and 2019. The rate of improvement was 1·2% per year in the 1970s, but this was followed by a reversal in the 1980s and 1990s, when PPD increased by 0·1% per year for two decades, erasing the gains from the 1970s. In the 2000s, PPD started to decline again, but at a slow rate of 0·6% per year. Currently, PPD of Nigeria (62·5%) is one of the highest in sub-Saharan Africa and is largely driven by the unfinished infectious disease agenda, worsening socioeconomic inequality, and the growing incidence of NCDs such as diabetes, hypertension, and cancers. Emerging challenges also include rising rates of poverty and conflict-related decreased access to health care.^20^

From 1970 until 2000, Mexico showed steady progress, with rate of improvement in PPD averaging between 1·2% and 2·0% per decade. However, gains stalled in the past two decades, and PPD has remained at around 30% since around 2003 (data not shown). A narrow set of three conditions—ischaemic heart disease, diabetes, and injuries resulting from interpersonal violence—accounted for the largest share of premature deaths, offsetting health gains in infectious diseases and explaining the poor performance. Increased mortality from ischaemic heart disease and diabetes has mainly affected the 50–69-year age group, whereas interpersonal violence is concentrated in ages 15–49 years.^21^, ^22^, ^23^, ^24^ Undertreated or untreated diabetes in adult Mexicans is the major challenge.^25^ At 75% in 2022, the combined prevalence of obesity and overweight among the adult population in Mexico is one of the highest in the world.^23^ An underfunded and fragmented primary health-care system has been unable to contain the rising prevalence of hypertension, dyslipidaemia, and high blood sugar concentrations over the past decades. On the other hand, the underlying determinants of deaths associated with violence remain a complex multifactorial agenda perceived to be beyond the traditional scope of health-policy intervention.

**Figure 4:**
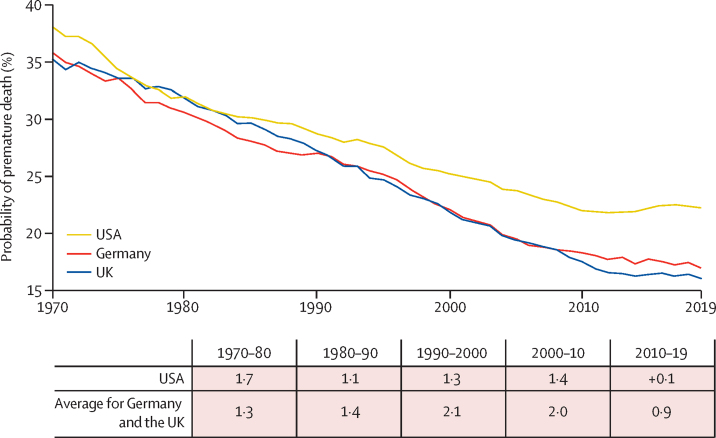
Country progress in PPD and average rates of improvement for USA versus Germany and the UK, 1970–2019, by decade

Adults in the USA have higher PPD and poorer health compared with their counterparts in other high-income nations. Although the peer nations of the USA continue to make strides in improving adult survival, the USA has witnessed a stark stagnation in such progress, particularly since the 1970s.^26^ PPD declined by an average of 2·0% annually in the 1970s, but subsequent decades saw this rate of decline halved, or even dissipated in the 2010s (figure 4). The stagnation disproportionately affects younger Americans. Deaths before age 50 years constitutes a substantial portion of the disparity in life expectancy between sexes in the USA and overall compared with other high-income nations.^26^ The trend is most pronounced among non-Hispanic White Americans, whose deaths comprise about two-thirds of all deaths in the USA since 1990 and who have seen slight improvement in reducing mortality since 1990. By contrast, mortality trends among Hispanic Americans have improved, driven partly by immigration.^27^ Among Black Americans, notable strides in reducing premature mortality have been seen.^28^ Among non-Hispanic White Americans, stagnation has occurred in those who have attained high-school education or less; those attending college continue to show overall decline in PPD.^29^ The COVID-19 pandemic amplified these marked educational differences.^30^ The phenomenon has been termed diseases of despair, and causes include increases in opioid-related deaths, cirrhosis, and suicides.^31^ However, the narrative is clearly incomplete, because rising mortality extends to vascular disease, chronic lung disease, injuries, and homicides. Analysis indicates that tobacco-related causes contributed to nearly half of the excess deaths among lower-educated non-Hispanic White Americans from 1990 to 2019, a finding consistent with international comparisons.^32^ Since 2010, the combined contribution of smoking, opioids, cirrhosis, suicide, and other external injuries has been approximately two-thirds of all excess deaths among non-Hispanic White Americans (Tang X, Daila Lana School of Public Health, University of Toronto, Canada, personal communication).

Countries with a rate of improvement in PPD better than 2·2% between 2010 and 2019 do not only include those with high PPD or high child mortality, but also include higher-income countries with low PPD and predominantly NCD-related mortality. Indeed, changes in PPD since 1970 for the world have largely been driven by improvements in ages 50–69 years. In 2010–19, about 50% of the improvements in PPD were attributable to this age group. If countries with a medium rate of improvement (between 2·2% and 1·0%) can achieve the same rate of improvement as their better-performing regional neighbours through benchmarking, halving premature death by 2050 is feasible but requires sustained and substantial investments.

Historically, countries that made the most progress in reducing PPD did so by implementing a limited set of interventions that addressed a relatively small number of diseases, injuries, and risk factors. For example, about a third of the gains in life expectancy in low-income countries between 2002 and 2019 were attributable to mortality reductions from treatment of HIV and AIDS, tuberculosis, and malaria.^33^ In sub-Saharan Africa, the overall decline in mortality has been substantial in the age group 0–14 years, and these deaths are easily preventable through cost-effective interventions. At the other end of the spectrum, nearly half of the reduction in cardiovascular mortality in the USA between 1980 and 2000 was attributable to reductions in tobacco use, high systolic blood pressure, and high cholesterol.^34^ Secondary prevention of cardiovascular disease, giving effective medicines to those who have experienced an event, can substantially reduce mortality.^35^ In some cases, mortality reductions can be substantial and rapid. For example, following a 1995 ban on organophosphate pesticides, suicide mortality in Sri Lanka declined by about 50% in the following decade.^36^ A prioritised approach to health conditions and interventions could allow countries with fewer resources to achieve considerable reductions in mortality at a reasonable cost.^37^, ^38^, ^39^ CIH3 concluded that 50 by 50 can be achieved by focusing on cost-effective interventions and policies targeting selected priority conditions, emphasis on tobacco cessation, and a redefined role for developmental assistance.^5^

Lastly, some of the most populous countries are unlikely to reach 50 by 50 on the basis of recent trends, even if most other countries could do so. These countries include Mexico, Viet Nam, Nigeria, and the USA. Among all countries, the number of countries from Latin America and the Caribbean in this category is of particular concern.

Although reducing the probability of premature death is a worthy goal for global health, people also care about living healthy lives. Prevalence of morbidity, the number of people living with chronic disease, the number of years they live in such conditions, and health-related quality of life at all ages are therefore of substantial interest. By reducing premature mortality, most people will live longer and healthier lives (for a more detailed discussion of the relationship between PPD, life expectancy, and healthy life expectancy [HALE], see appendix pp 2– 3). As shown by Salomon and colleagues,^40^ in countries where life expectancy has increased, the total number of years lived in good health (HALE) has also increased.^41^ However, as people live longer, the number of years lived with chronic disease will increase. This trend, combined with an ageing of the population, will lead to higher demand for long-term health services. Investments in services reducing chronic disease morbidity are therefore needed.

For our analysis, we relied on estimates from the UN WPP 2024, which are widely used and generally considered reliable. For about half of the countries, mortality rates were derived by UN WPP from vital registration systems. However, for the other half, they combined model life tables with censuses, surveys, and other demographic-input parameters.^42^ There is substantial uncertainty surrounding data on risks of dying in countries that do not have comprehensive death registration systems, especially for adult deaths. Levels of and trends in mortality should therefore be interpreted with caution. WPP 2024 does not provide uncertainty intervals for their estimates, so we could not produce the corresponding ranges.

Our study aimed to look at the feasibility of halving the probability of premature death by 2050, not the absolute number of premature deaths. The crude death rate is projected to increase towards 2050 because of demographic change, with many countries moving towards inverted population pyramids.^42^ Halving the absolute number of premature deaths will therefore be more difficult. Our justification for exploring halving PPD is that the probability of premature death is amenable to policies and health investments, whereas changes in population size and age distribution of populations are not.^43^ PPD is therefore a policy-relevant outcome and easier to communicate to decision makers and citizens than many other indicators.

Decomposing the relative contribution of leading causes of death to the rate of reduction of PPD can further help in identifying the most effective policies and interventions. We did not do so in this study; this is discussed more extensively in CIH3.^5^

In conclusion, we found that 50 by 50, a 50% reduction in the probability of premature death by 2050, is a feasible global goal that would substantially improve the chance of living a long and healthy life everywhere, with the caveat that this feasibility can only occur in areas free of war, other major effects of political disruption, natural disasters, or a major new epidemic that cannot be reliably predicted or their effects quantified. Substantial investments in both child and adult health are needed to sustain or accelerate the rate of improvement for high-performing and medium-performing countries. For low-performing countries, 50 by 50 might remain an aspiration. Historical evidence indicates that a limited set of interventions that address a relatively small number of diseases, injuries, and risk factors can substantially boost progress on reducing PPD. However, as people live longer, the absolute number of years lived with chronic disease will increase and so will demand for services to prevent, postpone, or ameliorate chronic morbidity.

### Contributors

### Data sharing

All data used in this article are available from UN World Population Prospects 2024.

## Declaration of interests

SB declares support from the University of Bergen, Bergen, Norway. AYC declares support for this work from the Norwegian Agency for Development Cooperation. DTJ declares support for the Commission from the Bill & Melinda Gates Foundation. WM declares support for this work from the Gates Foundation. OFN declares support for this work from the Norwegian Agency for Development Cooperation (Oslo, Norway) and a Norwegian Research Council Centre of Excellence grant (Oslo, Norway). OO declares support for this work from the Gates Foundation. DW declares salary support for participation in *The Lancet Commission on Investing in Health* (322282–SFF). GY declares research funding to his institution to support this work from the Gates Foundation. All other authors declare no competing interests.
