# Supplementary material for: Halving premature death and improving quality of life at all ages: cross-country analyses of past trends and future directions
Source: Lancet. 2024 Dec 14;404(10470):2437–46. doi: 10.1016/S0140-6736(24)02417-6 (PMC11667436; doi:10.1016/S0140-6736(24)02417-6)
Supplement: Supplementary appendix [file mmc1.pdf]

THE LANCET

Supplementary appendix

This appendix formed part of the original submission and has been peer reviewed. We post it as supplied by the authors.

Supplement to: Norheim OF, Chang AY, Bolongaita S, et al. Halving premature death and improving quality of life at all ages: cross-country analyses of past trends and future directions. *Lancet* 2024; published online Nov 21. https://doi.org/10.1016/S0140-6736(24)02417-6.

Appendix: Halving premature death and improving the quality of life at all ages: cross-country analyses of past trends and future directions

Ole F Norheim, Angela Y Chang, Sarah Bolongaita, Mariana Barraza-Lloréns, Ayodamope Fawole, Lia Tadesse Gebremedhin, Eduardo González-Pier, Prabhat Jha, Emily K Johnson, Omar Karlsson, Mizan Kiros, Sarah Lewington, Wenhui Mao, Osondu Ogbuoji, Muhammad Pate, Jennifer L Sargent, Xuyang Tang, David Watkins, Gavin Yamey, Dean T Jamison, Richard Peto.

Methods: 3
Decomposition of changes in PPD by age 3
Health-related quality of life and PPD 3
Sensitivity analysis with other age cut-off values for premature death 4
Figures 6
Figure A1. Regions as classified* by the Third Commission on Investing in Health (CIH3). 6
Figure A2. Probability of dying between ages 0 and 14, both sexes, 1970-2019, 30 most populous countries. 9
Figure A3. Scatterplot of level of probability of premature death (PPD) in 2019 and average annual rate of change between 2010-2019. *Data source: World Population Prospects 2024.*[6] 10
Figure A4. PPD by sex, 1970-2023: (a) USA and (b) Thailand. *Source: World Population Prospects 2024.*[6] 11
Figure A5: Decomposition of changes in PPD by age groups for the world and CIH3 regions, both sexes, by decade. *Data source: World Population Prospects 2024.*[6] 12
Figure A6. Life expectancy and healthy life expectancy (HALE) at birth, both sexes combined, in 2019. 13
Figure A7. Life expectancy (total column), healthy life years, and years lived with disability for top five and bottom five of the 30 most populous countries. Both sexes combined, 2019. 14
Tables 15
Table A1. High- and very low-performing countries in terms of average annual rate of improvement (AARI) for PPD in the period 2010-19, both sexes combined, by CIH3 region. Countries are listed alphabetically. *Source: World Population Prospects 2024.*[6] 15
Table A2. Halving premature death in 31 years: 37 countries that achieved halving of premature death in the last half century (over 31 years or less in the period 1970-2019). Both sexes combined. *Source: World Population Prospects 2024.*[6] 17
Table A3 Probability of premature death in 2019 for all countries and average annual rate of improvement (AARI) in the period 2010-19, by age group and sex. *Source: World Population Prospects 2024.*[6] 18
Table A4. Years lived with disability (YLD) and proportion of YLD over life expectancy. Calculated from healthy life expectancy (HALE) and life expectancy at birth in 2019 for the 30 most populous countries. Both sexes combined. *Source: WHO Global Health Observatory*[4] 25

Methods:

Decomposition of changes in PPD by age

To understand the contribution of the changes in the probability of death in each age group (0-14, 15-49, 50-69) to the changes in overall PPD (0-69), we decomposed the change in PPD for each decade by the contribution of the age groups:

Let $q_0 = {}_{70}q_0$

$q_1 = {}_{15}q_0$

$q_2 = {}_{35}q_{15}$

$q_3 = {}_{20}q_{50}$

By definition, $q_0 = 1 – (1-q_1)(1-q_2)(1-q_3)$. Then, the change in q_0 can be expressed as:

$$\Delta q_0 = \frac{\partial q_0}{\partial q_1}\Delta q_1 + \frac{\partial q_0}{\partial q_2}\Delta q_2 + \frac{\partial q_0}{\partial q_3}\Delta q_3$$

By taking the partial derivative of the equation above, we can calculate the contribution of each age group. For example, the contribution of Δq_1 to Δq_0 is $\frac{\frac{\partial q_0}{\partial q_1}\Delta q_1}{\Delta q_0}$.

Health-related quality of life and PPD

We here briefly discuss the relationship between probability of premature death (PPD), life expectancy (LE) and healthy life expectancy (HALE). Reducing PPD will improve life expectancy. Reducing mortality rates in younger age groups will improve life expectancy more than reducing mortality in older age groups.[1] HALE is a summary measure of population health promoted by the World Health Organization (WHO) and the Global Burden of Disease.[2-4] This indicator measures life expectancy adjusted for health-related quality of life in those years. As shown by Salomon and others, when life expectancy has increased for most countries in the past decades up to the Covid-19 pandemic, the number of years lived in good health (HALE) has also increased.[2,5]

As indicated in figures A1, A2, and Table A4, mortality change is the main driver for changes in HALE. Preventing risk factors and treating conditions do reduce premature mortality and

thus improve life expectancy and HALE. This means that by reducing PPD, people live longer and healthier lives. Figure A2 illustrates this relationship: the five countries with the highest HALE among the 30 most populous countries in 2019 could expect about 16-17 more healthy life years than the five countries with the lowest HALE. Progress leads to people living more life years in good health. Although the same top five countries could expect about 1-2 more years lived with disability compared to the bottom five countries, the total gain is much higher and the relative proportion (12-14%) between healthy years and years with disability is about the same (see Table A4).

By reducing premature mortality, most people will live longer and healthier lives. Yet, as people live longer, the absolute number of years lived with chronic disease will increase. This trend, combined with an inversion of the population pyramid, will lead to higher demand for health services, especially service that can reduce chronic morbidity.

Although mortality change is the main driver for changes in HALE, there are some exceptions in which diseases cause substantial health burden but do not result in high mortality. Musculoskeletal disorders such as back pain, neck pain, and osteoarthritis are nonfatal but contribute to a high burden of disease globally. Mental health disorders such as depression, anxiety, intellectual disability, alcohol use disorders, and attention deficit disorder also account for a substantial burden of disease combined.[5] Diabetes mellitus affects both mortality and morbidity, and prevalence has increased substantially since 1990.[5] These challenges must also be addressed.

Sensitivity analysis with other age cut-off values for premature death

We performed a sensitivity analysis for other age cut-off values for PPD. PPD values decrease when a lower age threshold (60 years) is used and increase when a higher age threshold (80 years) is used. Regional rates of improvement are faster with using $_{60}q_0$ and slower with $_{80}q_0$. The exception for this is the US: rates of improvement worsen when using a lower age threshold (+0.3%) and improve when using a higher age threshold (0.3%). The 2019 PPD rankings across regions don't change when using $_{60}q_0$ compared to $_{70}q_0$, but they

are slightly shuffled when using ${}_{80}q_0$. MENA (55%) had worse 2019 ${}_{80}q_0$ than WPSA (51%) and LAC (50%), and China (46%) performed worse than the US (41%).

At the country level, the prospect of achieving the “50 by 50” goal seems more optimistic when using ${}_{60}q_0$: 13 of the top 30 countries have a rate of improvement better than 2.2% with China, Japan, Spain, and India joining the group. The number of countries with rates of improvement below 1% decreases from 9 to 7. When using ${}_{80}q_0$, on the other hand, the goal seems far less feasible. Only Korea had a rate of improvement better than 2.2%. South Africa, which had a ${}_{70}q_0$ rate of improvement of 2.4%, fell substantially in the rankings with an ${}_{80}q_0$ rate of improvement of 0.9%.

Figures

Figure A1. Regions as classified* by the Third Commission on Investing in Health (CIH3).

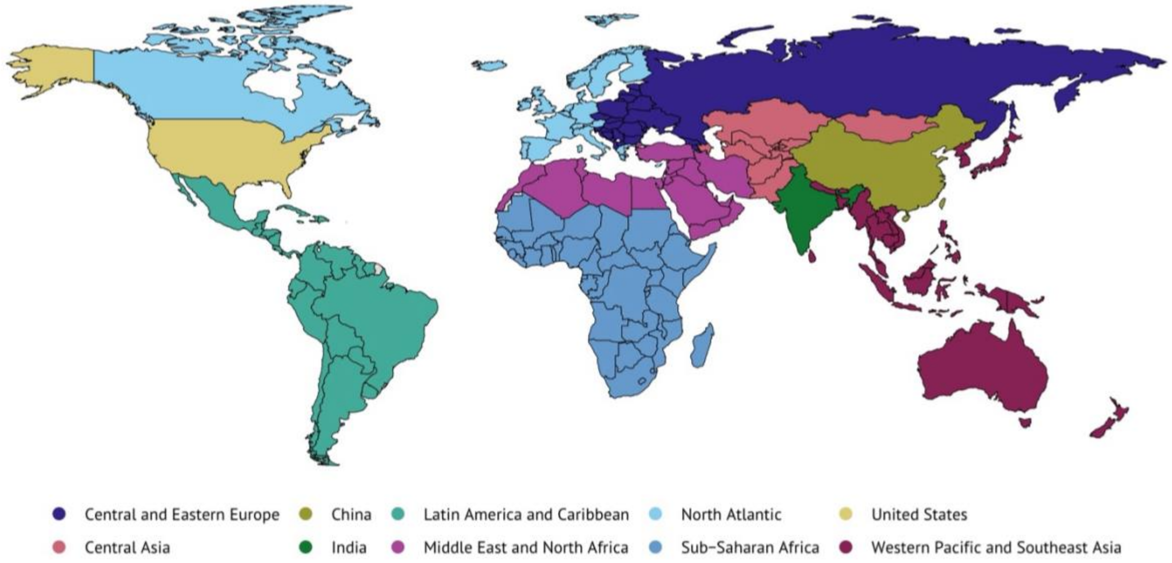

*** Locations by CIH region**

**Central Asia**		
Afghanistan	Azerbaijan	Kazakhstan
Kyrgyz Republic	Mongolia	Pakistan
Tajikistan	Turkmenistan	Uzbekistan
**Central and Eastern Europe**		
Albania	Armenia	Belarus
Bosnia and Herzegovina	Bulgaria	Croatia
Czech Republic	Estonia	Georgia
Hungary	Latvia	Lithuania
Moldova	Montenegro	North Macedonia
Poland	Romania	Russian Federation
Serbia	Slovak Republic	Slovenia
Ukraine		
**China**		
**India**		
**Latin America and Caribbean**		
Argentina	Bahamas, The	Belize

Bolivia	Brazil	Chile
Colombia	Costa Rica	Cuba
Dominican Republic	Ecuador	El Salvador
Guatemala	Guyana	Haiti
Honduras	Jamaica	Mexico
Nicaragua	Panama	Paraguay
Peru	Suriname	Trinidad and Tobago
Uruguay	Venezuela, RB	
**Middle East and North Africa**		
Algeria	Bahrain	Egypt, Arab Rep.
Iran, Islamic Rep.	Iraq	Israel
Jordan	Kuwait	Lebanon
Libya	Morocco	Oman
Qatar	Saudi Arabia	Syrian Arab Republic
Tunisia	Türkiye	United Arab Emirates
Yemen, Rep.		
**North Atlantic**		
Austria	Belgium	Canada
Cyprus	Denmark	Finland
France	Germany	Greece
Iceland	Ireland	Italy
Luxembourg	Malta	Netherlands
Norway	Portugal	Spain
Sweden	Switzerland	United Kingdom
**Sub-Saharan Africa**		
Angola	Benin	Botswana
Burkina Faso	Burundi	Cabo Verde
Cameroon	Central African Republic	Chad
Comoros	Congo, Dem. Rep.	Congo, Rep.
Côte d'Ivoire	Djibouti	Equatorial Guinea
Eritrea	Eswatini	Ethiopia
Gabon	Gambia, The	Ghana
Guinea	Guinea-Bissau	Kenya
Lesotho	Liberia	Madagascar
Malawi	Mali	Mauritania
Mauritius	Mozambique	Namibia
Niger	Nigeria	Rwanda
Senegal	Sierra Leone	Somalia

South Africa	South Sudan	Sudan
Tanzania	Togo	Uganda
Zambia	Zimbabwe	
**United States**		
**Western Pacific and Southeast Asia**		
Australia	Bangladesh	Bhutan
Brunei Darussalam	Cambodia	Fiji
Indonesia	Japan	Korea, Dem. People's Rep.
Korea, Rep.	Lao PDR	Malaysia
Maldives	Myanmar	Nepal
New Zealand	Papua New Guinea	Philippines
Singapore	Solomon Islands	Sri Lanka
Thailand	Timor-Leste	Vanuatu
Vietnam		

Notes: Countries were included in a CIH region if they were United Nations Member States with populations of at least 300 000 in 2022. For the CIH World region, if an input dataset contained a World region, those values were used for the CIH World region; if a dataset did not contain a World region, values for the CIH World region were calculated from all locations with available data, regardless of UN Member State status or population size.

Figure A2. Probability of dying between ages 0 and 14, both sexes, 1970-2019, 30 most populous countries.

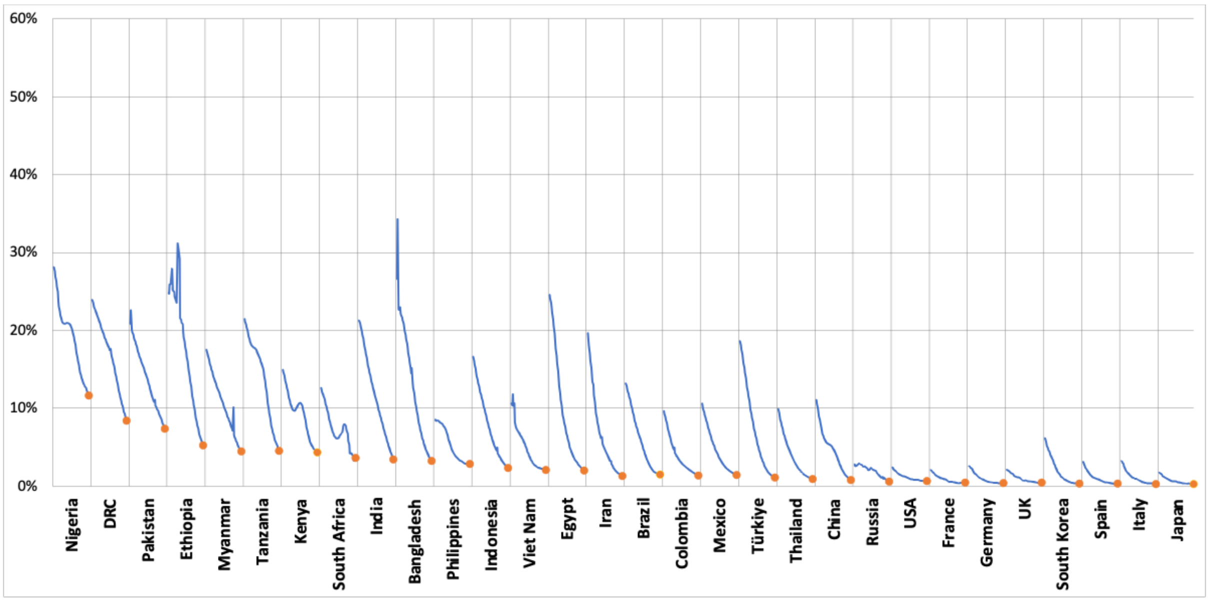

Figure A3. Scatterplot of level of probability of premature death (PPD) in 2019 and average annual rate of change between 2010-2019. *Data source: World Population Prospects 2024.*[6]

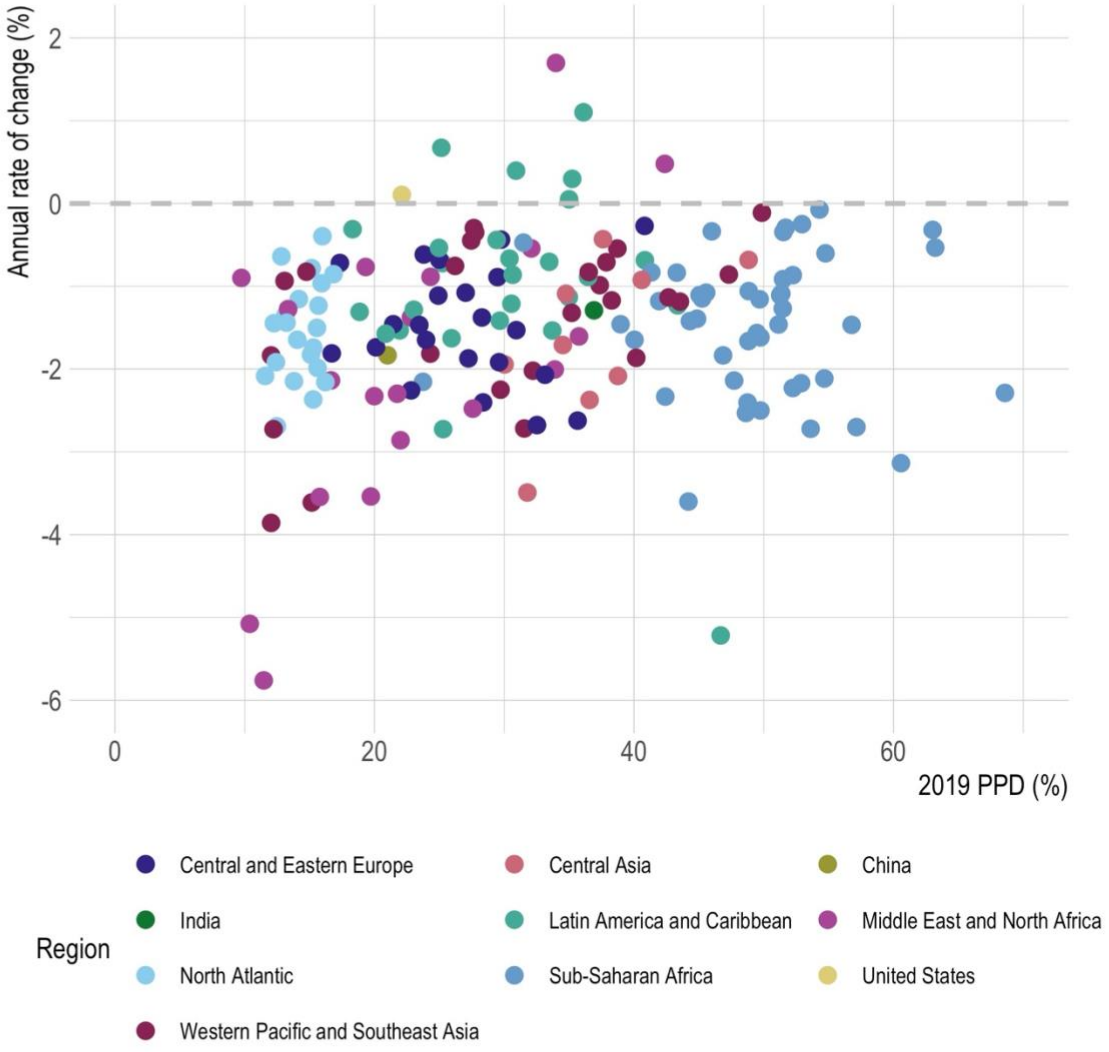

Note: Colour coded by CIH3 region. Horizontal dotted line at 0% annual rate of improvement.

Figure A4. PPD by sex, 1970-2023: (a) USA and (b) Thailand. *Source: World Population Prospects 2024.*[6]

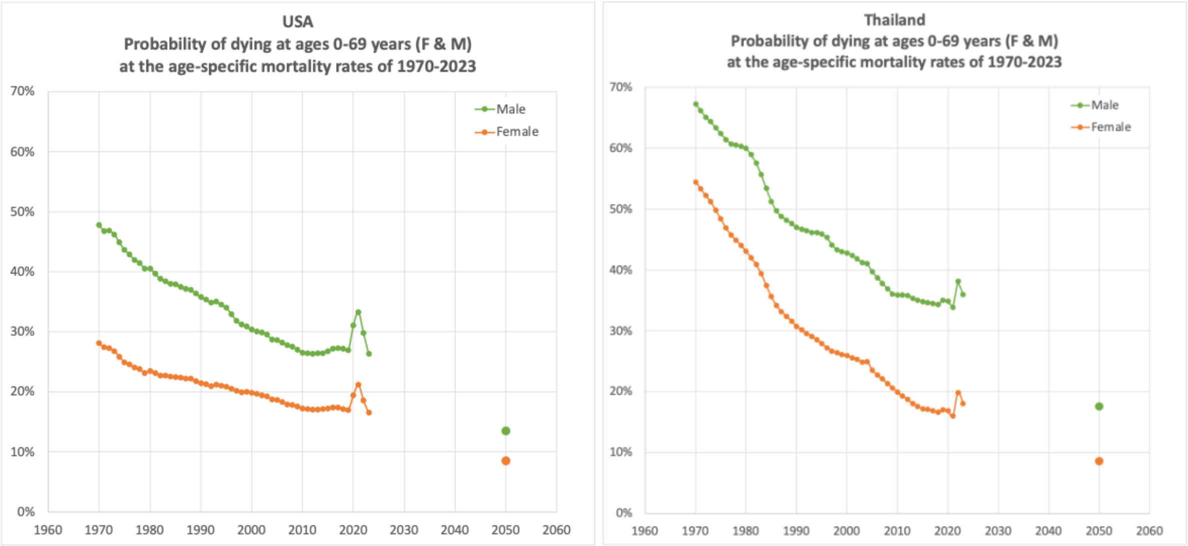

Note: The dots at year 2050 indicate PPD at 50% of the PPD in 2019 (baseline year), by sex.

Figure A5: Decomposition of changes in PPD by age groups for the world and CIH3 regions, both sexes, by decade. *Data source: World Population Prospects 2024.*[6]

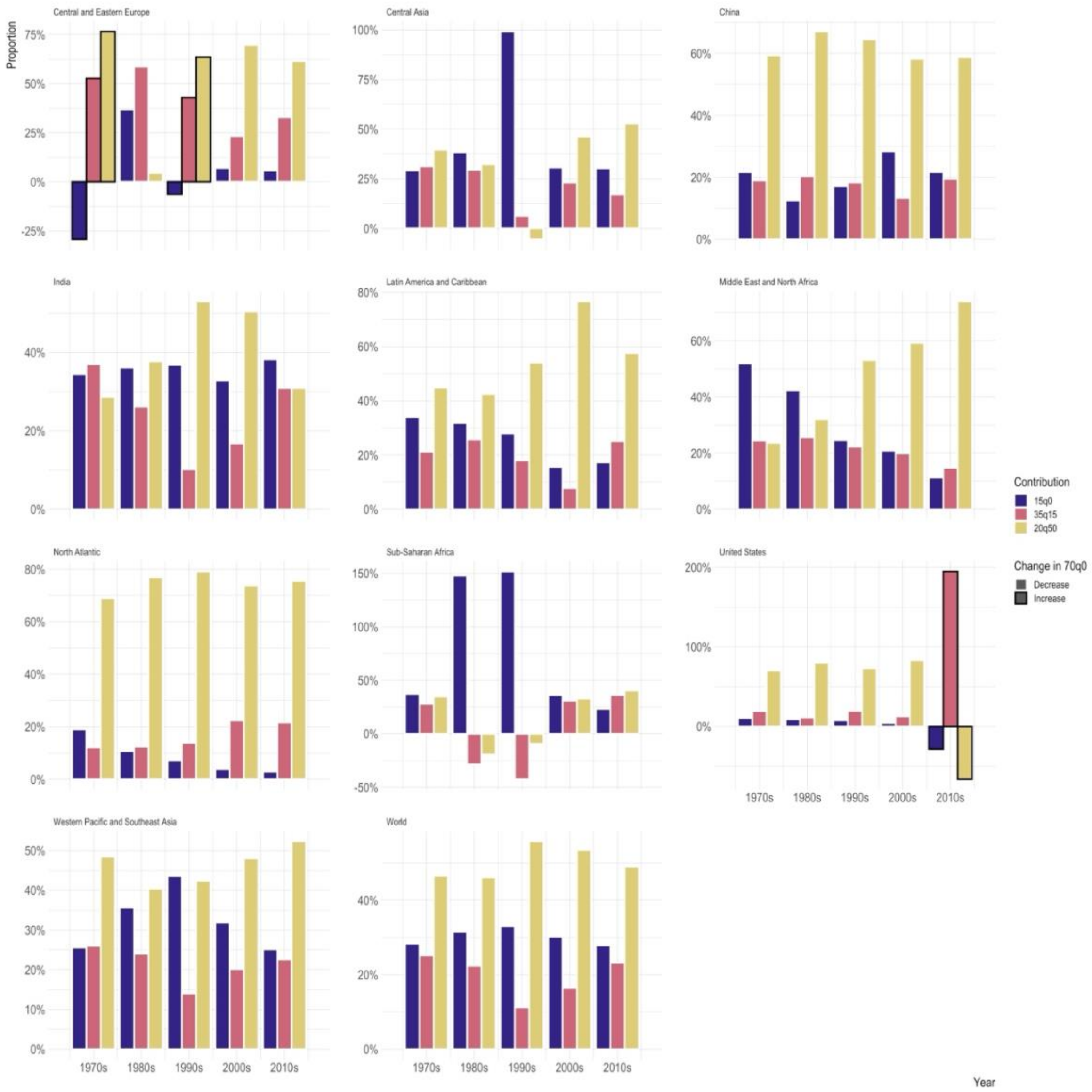

Figure A6. Life expectancy and healthy life expectancy (HALE) at birth, both sexes combined, in 2019.

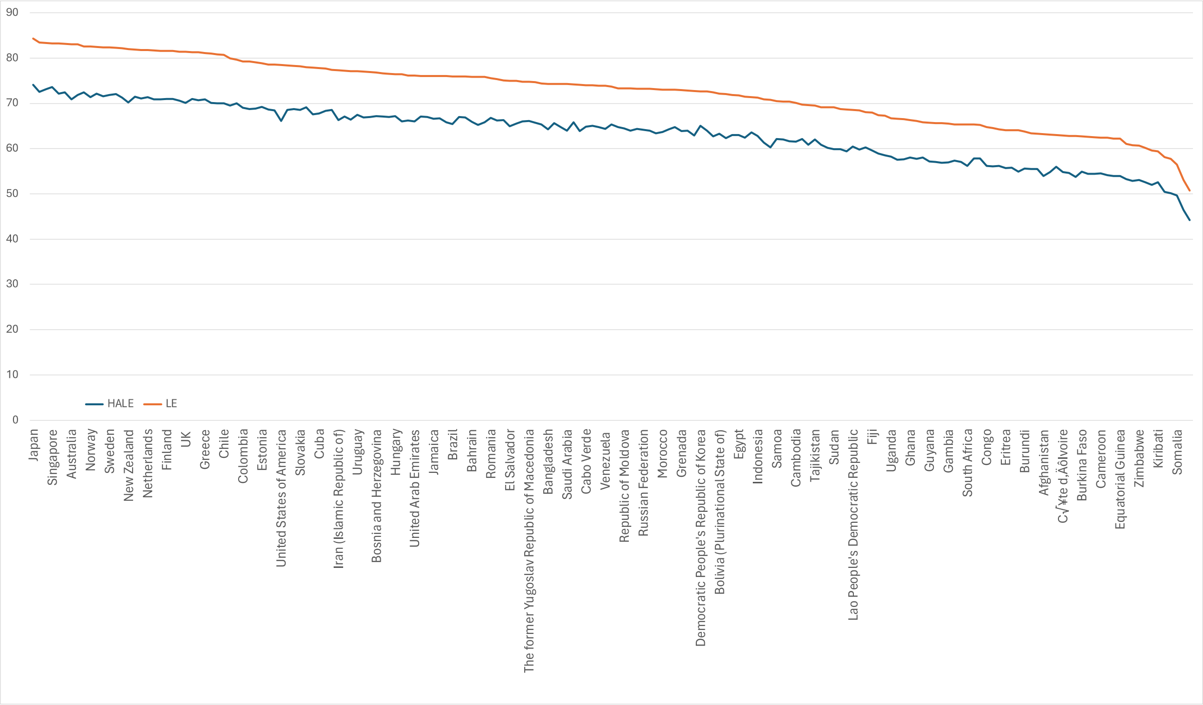

Note: Countries ranked from highest to lowest life expectancy. All countries of the world are represented in the graph, but not all names are listed. Data extracted from *WHO Global Health Observatory.*[4]

Figure A7. Life expectancy (total column), healthy life years, and years lived with disability for top five and bottom five of the 30 most populous countries. Both sexes combined, 2019.

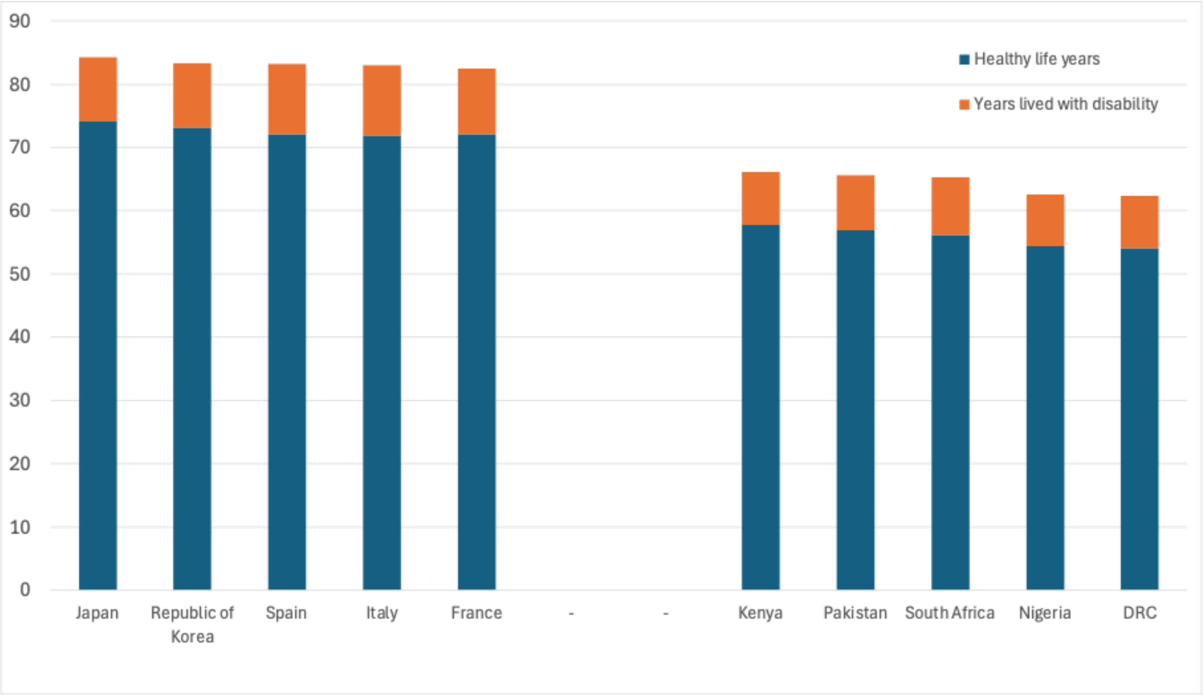

Note: Countries with high life expectancy have higher number of healthy life years (blue bars) compared to countries with lower life expectancy. DRC = Democratic Republic of Congo. *Source: WHO Global Health Observatory.*[4]

Tables

Table A1. High- and very low-performing countries in terms of average annual rate of improvement (AARI) for PPD in the period 2010-19, both sexes combined, by CIH3 region. Countries are listed alphabetically. *Source: World Population Prospects 2024.*[6]

CIH3 region	Population	Average annual rate of improvement in 2010-2019
**Central and Eastern Europe**	Belarus*	2.7%
	Estonia*	2.3%
	Lithuania*	2.4%
	Russian Federation*	2.7%
**Central Asia**	Kazakhstan*	3.6%
	Kyrgyzstan*	2.4%
**Latin America and Caribbean**	Haiti*	5.4%
	Peru*	2.8%
	Bahamas	+1.1%
	Cuba	+0.7%
	El Salvador	+0.1%
	Jamaica	+0.3%
	Venezuela	+0.4%
**Middle East and North Africa**	Iran*	2.4%
	Jordan*	2.9%
	Kuwait*	5.9%
	Morocco*	2.5%
	Oman*	3.6%
	Qatar*	5.2%
	Saudi Arabia*	3.6%
	State of Palestine*	2.2%
	Türkiye*	2.3%
	Syrian Arab Republic	+1.7%
	Yemen	+0.5%
**North Atlantic**	Finland*	2.4%
	Norway*	2.7%
**Sub-Saharan Africa**	Botswana*	3.7%
	Eswatini*	3.2%
	Ethiopia*	2.4%
	Lesotho*	2.3%
	Malawi*	2.6%

	Namibia*	2.8%
	Sierra Leone*	2.3%
	South Africa*	2.4%
	Zambia*	2.5%
	Zimbabwe*	2.7%
	Central African Republic	+2.2%
	Seychelles	+0.1%
**Western Pacific and Asia**	Bangladesh*	2.8%
	Dem. People's Republic of Korea*	2.3%
	Maldives*	3.7%
	Republic of Korea*	3.9%
	Singapore*	2.8%
**Other**	United States of America	+0.1%

* = Countries with Average Annual Rate of Improvement (AARI) better than 2.2% in the period 2010-19, listed by CIH region.

Table A2. Halving premature death in 31 years: 37 countries that achieved halving of premature death in the last half century (over 31 years or less in the period 1970-2019). Both sexes combined. *Source: World Population Prospects 2024.*[6]

	Initial year	Initial PPD
**Algeria**	1970	74%
**Armenia**	1988	65%
**Australia**	1970	38%
**Bahrain**	1991	30%
**Bangladesh**	1991	62%
**Bosnia and Herzegovina**	1992	70%
**Cabo Verde**	1988	49%
**Cambodia**	1975	100%
**Chile**	1970	50%
**China**	1970	60%
**Colombia**	1985	46%
**Cyprus**	1970	41%
**El Salvador**	1980	72%
**Guatemala**	1982	70%
**Iceland**	1974	30%
**Iran (Islamic Republic of)**	1983	56%
**Ireland**	1978	34%
**Israel**	1973	35%
**Italy**	1983	28%
**Japan**	1970	33%
**Kuwait**	1983	31%
**Lebanon**	1975	58%
**Luxembourg**	1977	35%
**Maldives**	1970	70%
**Malta**	1970	36%
**New Zealand**	1977	34%
**Norway**	1987	25%
**Oman**	1970	69%
**Qatar**	1974	42%
**Republic of Korea**	1992	34%
**Saudi Arabia**	1970	61%
**Singapore**	1970	43%
**Slovenia**	1983	36%
**Timor-Leste**	1978	95%
**Tunisia**	1970	60%
**United Arab Emirates**	1970	51%
**Viet Nam**	1972	66%

Table A3 Probability of premature death in 2019 for all countries and average annual rate of improvement (AARI) in the period 2010-19, by age group and sex. *Source: World Population Prospects 2024.*[6]

	Female								Male								Both sexes							
	Probability of death in 2019				AARI				Probability of death in 2019				AARI				Probability of death in 2019				AARI			
	0-14	15-49	50-69	0-69	0-14	15-49	50-69	0-69	0-14	15-49	50-69	0-69	0-14	15-49	50-69	0-69	0-14	15-49	50-69	0-69	0-14	15-49	50-69	0-69
**World**	5%	6%	17%	25%	-3.2%	-2.0%	-1.1%	-1.5%	5%	9%	27%	37%	-2.9%	-1.5%	-0.9%	-1.1%	5%	7%	22%	31%	-3.0%	-1.7%	-1.0%	-1.3%
**Afghanistan**	7%	11%	30%	42%	-4.1%	-2.5%	-1.1%	-1.5%	8%	20%	40%	56%	-3.6%	2.0%	-0.3%	-0.1%	7%	15%	35%	49%	-3.8%	0.3%	-0.7%	-0.7%
**Albania**	1%	2%	10%	13%	-3.0%	-1.6%	0.1%	-0.4%	1%	4%	18%	22%	-4.1%	-3.0%	-0.1%	-0.8%	1%	3%	14%	17%	-3.6%	-2.5%	-0.1%	-0.7%
**Algeria**	2%	3%	15%	20%	-2.2%	-2.0%	-1.7%	-1.7%	3%	4%	20%	26%	-2.1%	-2.2%	-1.0%	-1.2%	3%	4%	18%	23%	-2.1%	-2.1%	-1.3%	-1.4%
**Angola**	8%	11%	30%	43%	-5.5%	-4.1%	-1.6%	-2.2%	9%	16%	40%	55%	-5.2%	-2.4%	-0.7%	-1.2%	9%	13%	35%	49%	-5.3%	-3.1%	-1.1%	-1.7%
**Argentina**	1%	4%	16%	20%	-2.8%	-0.5%	0.1%	-0.2%	1%	6%	25%	31%	-3.1%	-1.5%	-1.0%	-1.1%	1%	5%	20%	25%	-3.0%	-1.1%	-0.6%	-0.7%
**Armenia**	1%	2%	15%	18%	-4.5%	-3.0%	-2.4%	-2.5%	2%	7%	33%	39%	-4.7%	-2.4%	-1.3%	-1.4%	1%	5%	23%	27%	-4.6%	-2.5%	-1.8%	-1.9%
**Australia**	0%	2%	8%	10%	-2.1%	-0.9%	-1.0%	-1.0%	1%	4%	13%	16%	-3.1%	-0.5%	-0.9%	-0.8%	0%	3%	10%	13%	-2.7%	-0.6%	-1.0%	-0.9%
**Austria**	0%	2%	9%	11%	-2.9%	-1.9%	-1.0%	-1.2%	1%	3%	16%	19%	-1.8%	-2.8%	-2.2%	-2.2%	0%	2%	13%	15%	-2.3%	-2.5%	-1.8%	-1.8%
**Azerbaijan**	2%	3%	17%	22%	-6.1%	-2.7%	-2.5%	-2.7%	3%	7%	32%	39%	-5.6%	-1.7%	-1.5%	-1.6%	2%	5%	24%	30%	-5.8%	-1.9%	-1.8%	-2.0%
**Bahamas**	4%	9%	21%	31%	8.6%	3.2%	1.2%	2.0%	4%	13%	30%	41%	7.0%	0.1%	0.3%	0.5%	4%	11%	25%	36%	7.8%	1.3%	0.7%	1.1%
**Bahrain**	1%	2%	13%	15%	-2.6%	-1.7%	-2.4%	-2.3%	1%	3%	14%	18%	-2.0%	-1.9%	-2.3%	-2.1%	1%	2%	14%	17%	-2.3%	-1.8%	-2.3%	-2.2%
**Bangladesh**	4%	5%	20%	27%	-5.0%	-2.7%	-3.1%	-3.0%	4%	7%	28%	35%	-4.8%	-2.0%	-2.7%	-2.5%	4%	6%	24%	32%	-4.9%	-2.3%	-2.9%	-2.8%
**Barbados**	1%	4%	13%	18%	-2.3%	-1.2%	-0.8%	-0.9%	1%	6%	22%	28%	-2.2%	-1.3%	-0.8%	-0.9%	1%	5%	18%	23%	-2.3%	-1.3%	-0.8%	-0.9%
**Belarus**	0%	4%	16%	19%	-6.1%	-3.7%	-2.6%	-2.8%	0%	11%	40%	47%	-7.6%	-5.2%	-2.2%	-2.4%	0%	7%	27%	33%	-7.0%	-4.9%	-2.4%	-2.7%
**Belgium**	0%	2%	10%	12%	-0.1%	-3.1%	-1.4%	-1.6%	1%	3%	16%	19%	-1.9%	-2.7%	-2.3%	-2.2%	0%	3%	13%	16%	-1.2%	-2.8%	-1.9%	-2.0%
**Belize**	1%	5%	22%	26%	-4.1%	-2.7%	-1.7%	-1.8%	2%	9%	36%	43%	-4.0%	-1.4%	-0.5%	-0.6%	2%	7%	29%	35%	-4.0%	-1.8%	-1.0%	-1.1%
**Benin**	11%	16%	32%	49%	-2.0%	-0.4%	-0.2%	-0.5%	12%	18%	36%	54%	-1.7%	0.1%	0.1%	-0.2%	12%	17%	34%	52%	-1.8%	-0.2%	0.0%	-0.3%
**Bhutan**	3%	5%	21%	27%	-5.0%	-4.1%	-2.1%	-2.5%	3%	6%	30%	37%	-4.8%	-3.6%	-1.3%	-1.7%	3%	6%	26%	32%	-4.9%	-3.8%	-1.7%	-2.0%
**Bolivia**	5%	8%	26%	35%	-3.1%	-1.1%	-0.5%	-0.9%	6%	13%	34%	46%	-2.6%	-0.7%	-0.4%	-0.5%	6%	10%	30%	41%	-2.8%	-0.9%	-0.4%	-0.7%

**Bosnia and Herzegovina**	1%	2%	15%	17%	-1.8%	-0.7%	-0.2%	-0.3%		1%	4%	27%	31%	-2.0%	-0.9%	-0.9%	-0.9%		1%	3%	21%	24%	-1.9%	-0.7%	-0.6%	-0.6%
**Botswana**	5%	10%	28%	38%	-3.7%	-8.7%	-3.9%	-4.2%		5%	14%	38%	50%	-3.6%	-7.7%	-3.0%	-3.3%		5%	12%	33%	44%	-3.6%	-8.1%	-3.4%	-3.7%
**Brazil**	2%	4%	15%	19%	-2.6%	-1.9%	-1.7%	-1.7%		2%	9%	24%	33%	-2.8%	-2.3%	-1.4%	-1.5%		2%	7%	19%	26%	-2.7%	-2.2%	-1.6%	-1.6%
**Brunei Darussalam**	1%	4%	18%	22%	0.0%	-0.6%	-0.2%	-0.3%		1%	6%	27%	33%	0.1%	-0.4%	-0.2%	-0.2%		1%	5%	23%	28%	0.1%	-0.5%	-0.3%	-0.3%
**Bulgaria**	1%	3%	16%	19%	-4.0%	-1.5%	-0.3%	-0.6%		1%	7%	34%	40%	-6.4%	-1.0%	-0.2%	-0.4%		1%	5%	25%	30%	-5.4%	-1.1%	-0.2%	-0.4%
**Burkina Faso**	10%	14%	31%	47%	-3.6%	-1.9%	-1.0%	-1.4%		11%	18%	40%	56%	-3.3%	-1.2%	-0.7%	-0.9%		11%	16%	35%	51%	-3.4%	-1.5%	-0.8%	-1.1%
**Burundi**	8%	15%	31%	46%	-5.0%	-2.9%	-1.6%	-1.9%		9%	19%	37%	53%	-4.4%	-1.9%	-1.1%	-1.3%		9%	17%	34%	49%	-4.7%	-2.3%	-1.3%	-1.6%
**Cabo Verde**	1%	3%	12%	16%	-6.2%	-3.9%	-1.5%	-2.3%		2%	8%	24%	32%	-6.2%	-3.6%	-2.1%	-2.4%		2%	6%	18%	24%	-6.2%	-3.5%	-1.6%	-2.2%
**Cambodia**	3%	8%	22%	30%	-5.6%	-2.8%	-1.3%	-1.9%		4%	12%	30%	41%	-4.5%	-1.2%	-0.6%	-0.9%		4%	10%	25%	35%	-5.0%	-1.8%	-0.9%	-1.3%
**Cameroon**	8%	13%	32%	46%	-4.1%	-3.9%	-1.6%	-2.0%		10%	18%	41%	56%	-3.8%	-2.1%	-0.7%	-1.1%		9%	15%	36%	51%	-4.0%	-2.9%	-1.1%	-1.5%
**Canada**	1%	2%	9%	12%	-1.4%	0.1%	-1.1%	-0.9%		1%	4%	14%	18%	-1.2%	0.5%	-1.0%	-0.7%		1%	3%	12%	15%	-1.3%	0.4%	-1.1%	-0.8%
**Central African Republic**	30%	39%	66%	85%	6.2%	2.3%	2.7%	1.8%		32%	77%	81%	97%	5.9%	7.1%	3.7%	2.1%		31%	62%	74%	93%	6.0%	5.9%	3.2%	2.2%
**Chad**	16%	23%	39%	60%	-2.5%	-1.0%	-0.7%	-0.8%		17%	27%	45%	66%	-2.1%	-0.2%	-0.1%	-0.3%		16%	25%	42%	63%	-2.3%	-0.6%	-0.4%	-0.5%
**Chile**	1%	3%	12%	15%	-3.3%	-1.0%	-1.3%	-1.3%		1%	5%	18%	23%	-3.4%	-1.7%	-1.3%	-1.3%		1%	4%	15%	19%	-3.4%	-1.4%	-1.3%	-1.3%
**China**	1%	3%	12%	15%	-7.4%	-2.7%	-2.2%	-2.5%		1%	5%	22%	27%	-7.4%	-2.4%	-1.0%	-1.4%		1%	4%	17%	21%	-7.4%	-2.5%	-1.4%	-1.9%
**China, Taiwan Province of China**	1%	2%	9%	12%	-2.2%	-1.5%	-1.8%	-1.7%		1%	6%	20%	25%	-2.8%	-2.0%	-0.9%	-1.1%		1%	4%	14%	18%	-2.5%	-1.9%	-1.2%	-1.4%
**Colombia**	1%	4%	13%	17%	-2.8%	-0.7%	-1.4%	-1.3%		2%	8%	19%	27%	-3.1%	-2.5%	-1.4%	-1.6%		2%	6%	16%	22%	-3.0%	-2.0%	-1.4%	-1.5%
**Comoros**	6%	9%	29%	39%	-3.5%	-2.6%	-1.2%	-1.5%		7%	12%	40%	51%	-3.3%	-1.8%	-0.5%	-0.8%		6%	11%	34%	45%	-3.4%	-2.1%	-0.8%	-1.1%
**Congo**	6%	15%	34%	47%	-1.7%	-1.4%	-0.7%	-0.8%		7%	17%	43%	56%	-2.4%	-0.6%	0.5%	0.0%		6%	16%	38%	52%	-2.1%	-1.0%	-0.1%	-0.3%
**Costa Rica**	1%	3%	11%	14%	-2.4%	0.0%	-0.1%	-0.2%		1%	6%	16%	23%	-2.5%	-0.1%	-0.3%	-0.3%		1%	5%	13%	18%	-2.5%	0.0%	-0.3%	-0.3%
**Croatia**	1%	2%	11%	14%	-2.1%	-2.2%	-1.4%	-1.5%		1%	5%	25%	29%	-2.9%	-2.4%	-1.4%	-1.5%		1%	3%	18%	21%	-2.6%	-2.3%	-1.4%	-1.5%
**Cuba**	1%	3%	17%	20%	-0.3%	-0.4%	0.3%	0.2%		1%	5%	26%	31%	0.6%	0.1%	1.3%	1.0%		1%	4%	21%	25%	0.2%	-0.1%	0.9%	0.7%
**Cyprus**	0%	1%	9%	10%	-1.0%	-2.7%	-0.4%	-0.7%		0%	2%	16%	18%	-1.7%	-3.0%	-1.2%	-1.4%		0%	2%	12%	14%	-1.4%	-2.9%	-1.0%	-1.2%
**Czechia**	0%	2%	11%	14%	-1.4%	-1.0%	-1.7%	-1.5%		0%	4%	23%	27%	-0.8%	-2.0%	-2.0%	-1.9%		0%	3%	17%	20%	-1.1%	-1.7%	-1.9%	-1.8%
**Côte d'Ivoire**	8%	17%	38%	53%	-3.5%	-3.9%	-1.5%	-1.8%		10%	20%	45%	60%	-3.3%	-2.9%	-1.0%	-1.2%		9%	18%	42%	57%	-3.4%	-3.3%	-1.3%	-1.5%
**Dem. People's Republic of Korea**	2%	5%	17%	23%	-4.8%	-1.3%	-2.7%	-2.4%		3%	10%	28%	37%	-4.2%	-0.7%	-2.9%	-2.3%		2%	8%	22%	30%	-4.4%	-0.9%	-2.7%	-2.3%
**Democratic Republic of the Congo**	10%	15%	31%	47%	-3.3%	-2.0%	-1.1%	-1.4%		12%	19%	37%	55%	-3.0%	-1.2%	-0.7%	-0.9%		11%	17%	34%	51%	-3.1%	-1.6%	-0.9%	-1.1%
**Denmark**	0%	2%	11%	13%	-1.8%	-4.1%	-2.3%	-2.4%		0%	3%	17%	20%	-0.8%	-3.8%	-1.8%	-2.0%		0%	2%	14%	16%	-1.2%	-3.9%	-2.0%	-2.2%
**Djibouti**	7%	11%	27%	40%	-3.5%	-2.7%	-1.4%	-1.7%		9%	16%	34%	50%	-3.0%	-1.9%	-1.0%	-1.2%		8%	14%	31%	45%	-3.2%	-2.2%	-1.2%	-1.4%

**Dominica**	3%	4%	19%	25%	2.5%	-2.0%	-0.5%	-0.4%		4%	8%	34%	41%	2.8%	-0.3%	-0.1%	0.0%		3%	6%	27%	34%	2.7%	-0.9%	-0.1%	-0.1%
**Dominican Republic**	3%	5%	17%	24%	-0.5%	-1.4%	-1.6%	-1.3%		4%	10%	27%	37%	-0.9%	-1.7%	-1.2%	-1.1%		4%	8%	22%	31%	-0.7%	-1.6%	-1.3%	-1.2%
**Ecuador**	1%	4%	14%	19%	-3.6%	-2.1%	-0.8%	-1.2%		2%	8%	19%	27%	-3.8%	-2.3%	-0.9%	-1.3%		2%	6%	17%	23%	-3.7%	-2.2%	-0.9%	-1.3%
**Egypt**	2%	4%	25%	29%	-3.7%	-2.5%	-1.5%	-1.7%		3%	6%	37%	42%	-3.5%	-2.7%	-1.5%	-1.6%		2%	5%	31%	36%	-3.6%	-2.6%	-1.5%	-1.6%
**El Salvador**	1%	5%	20%	26%	-3.7%	-1.7%	0.4%	-0.2%		2%	17%	33%	46%	-3.9%	-1.0%	1.3%	0.3%		2%	11%	26%	35%	-3.8%	-1.2%	0.8%	0.1%
**Equatorial Guinea**	9%	12%	32%	45%	-3.7%	-2.8%	-1.1%	-1.5%		10%	15%	39%	53%	-3.4%	-1.9%	-0.6%	-1.0%		9%	14%	36%	50%	-3.6%	-2.2%	-0.8%	-1.2%
**Eritrea**	5%	10%	25%	36%	-4.1%	-3.1%	-1.7%	-2.0%		6%	14%	31%	45%	-3.4%	-2.2%	-1.1%	-1.3%		6%	12%	28%	40%	-3.7%	-2.6%	-1.4%	-1.7%
**Estonia**	0%	3%	11%	13%	-7.8%	-0.7%	-3.1%	-2.7%		0%	6%	29%	33%	-5.7%	-4.9%	-1.7%	-2.1%		0%	4%	19%	23%	-6.5%	-3.8%	-2.0%	-2.3%
**Eswatini**	6%	20%	39%	54%	-4.9%	-8.8%	-4.1%	-4.0%		7%	28%	51%	67%	-4.8%	-6.1%	-2.7%	-2.5%		7%	24%	45%	61%	-4.8%	-7.3%	-3.4%	-3.2%
**Ethiopia**	6%	10%	25%	36%	-6.1%	-5.2%	-2.7%	-3.2%		8%	16%	34%	49%	-4.7%	-2.7%	-1.4%	-1.7%		7%	13%	29%	42%	-5.3%	-3.7%	-2.0%	-2.4%
**Fiji**	3%	8%	37%	44%	1.0%	-1.0%	0.2%	0.0%		3%	10%	49%	56%	1.2%	-1.5%	-0.1%	-0.2%		3%	9%	43%	50%	1.1%	-1.3%	0.0%	-0.1%
**Finland**	0%	2%	8%	11%	-1.6%	-1.6%	-1.6%	-1.6%		0%	4%	16%	20%	-1.6%	-2.9%	-2.9%	-2.7%		0%	3%	12%	15%	-1.6%	-2.5%	-2.5%	-2.4%
**France**	0%	2%	9%	11%	-0.3%	-2.0%	-0.5%	-0.7%		1%	4%	17%	21%	0.3%	-2.5%	-1.3%	-1.4%		1%	3%	13%	16%	0.0%	-2.3%	-1.1%	-1.2%
**Gabon**	5%	9%	28%	37%	-4.1%	-2.7%	-0.9%	-1.4%		5%	13%	38%	49%	-4.2%	-1.2%	0.0%	-0.5%		5%	11%	33%	43%	-4.2%	-1.7%	-0.4%	-0.8%
**Gambia**	7%	13%	29%	42%	-3.9%	-1.9%	-1.0%	-1.4%		8%	16%	34%	49%	-3.4%	-1.3%	-0.7%	-1.0%		7%	14%	31%	45%	-3.6%	-1.6%	-0.9%	-1.2%
**Georgia**	1%	3%	16%	19%	-4.7%	-2.7%	-2.3%	-2.3%		1%	10%	37%	44%	-4.6%	-2.9%	-0.8%	-1.1%		1%	6%	25%	31%	-4.6%	-2.7%	-1.3%	-1.5%
**Germany**	0%	2%	10%	12%	-1.2%	-1.8%	-0.3%	-0.6%		1%	3%	18%	21%	-1.0%	-2.1%	-0.9%	-1.0%		0%	2%	14%	17%	-1.1%	-2.0%	-0.7%	-0.9%
**Ghana**	6%	13%	28%	41%	-4.1%	-2.3%	-1.2%	-1.5%		8%	17%	35%	50%	-3.4%	-0.9%	-0.5%	-0.7%		7%	15%	31%	46%	-3.7%	-1.5%	-0.8%	-1.1%
**Greece**	0%	1%	9%	10%	-0.4%	-2.5%	0.3%	-0.1%		0%	3%	19%	22%	-0.8%	-3.3%	0.1%	-0.4%		0%	2%	14%	16%	-0.7%	-3.1%	0.0%	-0.4%
**Guatemala**	3%	6%	22%	29%	-3.7%	-1.2%	0.1%	-0.4%		3%	12%	28%	38%	-3.8%	-3.0%	0.1%	-0.9%		3%	9%	25%	33%	-3.8%	-2.4%	0.1%	-0.7%
**Guinea**	12%	16%	32%	49%	-2.0%	-1.5%	-0.8%	-1.0%		14%	17%	36%	54%	-2.0%	-1.4%	-0.8%	-0.9%		13%	16%	33%	51%	-2.0%	-1.5%	-0.8%	-0.9%
**Guinea-Bissau**	9%	13%	31%	45%	-4.2%	-3.6%	-1.5%	-1.9%		10%	17%	40%	55%	-4.0%	-2.6%	-1.0%	-1.3%		9%	15%	35%	50%	-4.1%	-3.0%	-1.3%	-1.6%
**Guyana**	3%	7%	27%	34%	-2.8%	-2.6%	-1.8%	-1.8%		4%	14%	42%	52%	-2.8%	-1.8%	-0.6%	-0.8%		3%	11%	35%	43%	-2.8%	-2.1%	-1.2%	-1.2%
**Haiti**	6%	9%	28%	39%	-13.6%	-14.3%	-6.1%	-7.0%		8%	15%	40%	54%	-10.7%	-8.8%	-3.7%	-4.1%		7%	12%	34%	47%	-12.1%	-11.2%	-4.8%	-5.4%
**Honduras**	2%	6%	21%	27%	-3.9%	-3.1%	-1.7%	-2.0%		2%	8%	33%	40%	-3.7%	-2.0%	-1.1%	-1.2%		2%	7%	27%	34%	-3.8%	-2.4%	-1.4%	-1.5%
**Hungary**	0%	2%	17%	19%	-3.6%	-3.3%	-0.7%	-1.1%		1%	5%	34%	38%	-2.0%	-4.8%	-1.3%	-1.5%		1%	4%	25%	28%	-2.7%	-4.3%	-1.0%	-1.4%
**Iceland**	0%	2%	8%	10%	-15.6%	-0.4%	-0.4%	-0.7%		0%	3%	12%	16%	-2.4%	-2.0%	-0.3%	-0.7%		0%	3%	10%	13%	-7.0%	-1.4%	-0.3%	-0.6%
**India**	4%	6%	26%	33%	-6.1%	-3.1%	-0.2%	-1.2%		4%	9%	32%	41%	-5.6%	-2.7%	-0.9%	-1.4%		4%	7%	29%	37%	-5.8%	-2.8%	-0.6%	-1.3%
**Indonesia**	3%	7%	24%	32%	-3.9%	-1.9%	-1.2%	-1.4%		3%	9%	35%	43%	-3.7%	-1.2%	-0.6%	-0.8%		3%	8%	30%	37%	-3.8%	-1.5%	-0.8%	-1.0%

**Iran**	2%	3%	12%	16%	-3.8%	-3.5%	-2.6%	-2.7%		2%	6%	17%	24%	-3.8%	-2.8%	-1.9%	-2.1%		2%	5%	15%	20%	-3.8%	-3.0%	-2.2%	-2.4%
**Iraq**	3%	6%	23%	29%	-3.6%	-3.7%	-1.7%	-2.0%		3%	8%	32%	39%	-3.7%	-4.3%	-1.7%	-2.0%		3%	7%	27%	34%	-3.7%	-4.0%	-1.7%	-2.0%
**Ireland**	0%	2%	9%	11%	-1.9%	-2.0%	-2.3%	-2.2%		0%	3%	14%	17%	-2.2%	-3.3%	-2.0%	-2.1%		0%	2%	11%	14%	-2.1%	-2.9%	-2.1%	-2.2%
**Israel**	0%	1%	8%	10%	-3.9%	-1.4%	-1.3%	-1.4%		0%	3%	14%	17%	-2.4%	-2.1%	-1.1%	-1.2%		0%	2%	11%	13%	-3.1%	-1.9%	-1.1%	-1.3%
**Italy**	0%	1%	8%	9%	-2.9%	-1.6%	-0.9%	-1.0%		0%	3%	13%	16%	-2.7%	-2.2%	-1.6%	-1.7%		0%	2%	10%	12%	-2.8%	-2.0%	-1.4%	-1.5%
**Jamaica**	2%	5%	23%	29%	0.4%	0.7%	0.4%	0.4%		3%	9%	34%	42%	0.1%	0.8%	0.3%	0.3%		2%	7%	28%	35%	0.2%	0.9%	0.2%	0.3%
**Japan**	0%	2%	6%	8%	-1.6%	-2.1%	-1.4%	-1.5%		0%	3%	13%	16%	-2.8%	-3.1%	-1.9%	-2.0%		0%	2%	10%	12%	-2.3%	-2.8%	-1.7%	-1.9%
**Jordan**	2%	3%	13%	17%	-3.0%	-3.9%	-3.5%	-3.4%		2%	4%	22%	27%	-3.2%	-4.5%	-2.4%	-2.6%		2%	3%	18%	22%	-3.1%	-4.3%	-2.9%	-2.9%
**Kazakhstan**	1%	4%	17%	21%	-6.8%	-5.1%	-3.4%	-3.6%		1%	11%	35%	43%	-7.0%	-6.3%	-3.1%	-3.3%		1%	8%	25%	32%	-6.9%	-6.0%	-3.2%	-3.6%
**Kenya**	5%	17%	36%	49%	-2.3%	-1.7%	-0.9%	-1.0%		6%	21%	46%	60%	-2.5%	-0.8%	-0.1%	-0.3%		5%	19%	41%	55%	-2.4%	-1.2%	-0.4%	-0.6%
**Kuwait**	1%	1%	8%	10%	-1.8%	-4.1%	-5.5%	-4.9%		1%	3%	8%	12%	-2.0%	-1.6%	-8.5%	-6.5%		1%	2%	8%	11%	-1.9%	-2.2%	-7.4%	-5.9%
**Kyrgyzstan**	2%	5%	20%	25%	-5.0%	-3.7%	-2.9%	-3.0%		2%	11%	39%	47%	-4.5%	-3.9%	-2.1%	-2.1%		2%	8%	30%	37%	-4.7%	-3.9%	-2.3%	-2.4%
**Lao People's Democratic Republic**	5%	7%	26%	34%	-4.9%	-4.2%	-1.6%	-2.2%		6%	10%	36%	46%	-4.5%	-3.8%	-1.3%	-1.7%		5%	9%	31%	40%	-4.7%	-4.0%	-1.4%	-1.9%
**Latvia**	1%	4%	15%	18%	-5.0%	-1.7%	-2.3%	-2.2%		1%	10%	35%	42%	-7.1%	-2.6%	-1.9%	-1.8%		1%	7%	24%	30%	-6.1%	-2.3%	-1.9%	-1.9%
**Lebanon**	2%	3%	12%	15%	1.3%	-1.1%	-1.0%	-0.8%		2%	3%	20%	23%	1.4%	-1.4%	-1.1%	-0.9%		2%	3%	16%	19%	1.3%	-1.3%	-0.9%	-0.8%
**Lesotho**	8%	26%	46%	64%	-2.6%	-6.0%	-3.0%	-2.6%		10%	33%	57%	74%	-2.5%	-5.5%	-2.4%	-2.0%		9%	30%	51%	69%	-2.6%	-5.7%	-2.8%	-2.3%
**Liberia**	8%	14%	36%	50%	-2.9%	-0.2%	0.3%	-0.2%		10%	16%	42%	56%	-2.7%	-0.4%	0.1%	-0.3%		9%	15%	39%	53%	-2.8%	-0.3%	0.1%	-0.2%
**Libya**	1%	5%	19%	24%	-2.7%	-1.6%	-1.2%	-1.3%		2%	10%	31%	39%	-2.9%	2.5%	-0.4%	0.0%		2%	8%	25%	32%	-2.8%	1.1%	-0.8%	-0.5%
**Lithuania**	0%	3%	14%	17%	-5.3%	-2.6%	-2.2%	-2.2%		1%	9%	34%	41%	-4.7%	-4.6%	-2.2%	-2.4%		1%	6%	23%	28%	-4.9%	-4.0%	-2.2%	-2.4%
**Luxembourg**	1%	3%	9%	12%	2.2%	-2.4%	-2.0%	-1.6%		1%	3%	15%	18%	4.7%	-4.9%	-1.7%	-1.9%		1%	3%	12%	15%	3.1%	-3.8%	-1.7%	-1.8%
**Madagascar**	8%	13%	28%	43%	-0.9%	-0.7%	-0.3%	-0.4%		9%	16%	34%	49%	-0.8%	-0.4%	-0.2%	-0.3%		9%	14%	31%	46%	-0.9%	-0.5%	-0.3%	-0.3%
**Malawi**	5%	11%	30%	40%	-6.5%	-7.5%	-3.4%	-3.8%		6%	19%	44%	57%	-6.3%	-3.5%	-1.2%	-1.6%		5%	15%	36%	49%	-6.4%	-5.1%	-2.2%	-2.6%
**Malaysia**	1%	4%	17%	21%	-0.4%	0.0%	-0.6%	-0.5%		1%	7%	27%	33%	-0.4%	-0.5%	-0.4%	-0.4%		1%	6%	22%	27%	-0.4%	-0.3%	-0.5%	-0.4%
**Maldives**	1%	2%	10%	12%	-7.5%	-2.8%	-4.3%	-4.2%		1%	2%	15%	18%	-8.9%	-1.2%	-3.5%	-3.5%		1%	2%	13%	15%	-8.3%	-1.5%	-3.8%	-3.7%
**Mali**	12%	14%	32%	49%	-2.8%	-1.5%	-0.8%	-1.1%		13%	17%	39%	56%	-2.7%	-0.8%	-0.5%	-0.7%		12%	16%	36%	52%	-2.8%	-1.1%	-0.6%	-0.9%
**Malta**	1%	2%	8%	11%	4.0%	1.4%	-1.5%	-0.6%		1%	2%	13%	16%	-0.8%	-5.1%	-1.7%	-2.0%		1%	2%	10%	13%	1.9%	-2.6%	-1.5%	-1.5%
**Mauritania**	5%	8%	27%	36%	-3.0%	-0.7%	-0.5%	-0.7%		5%	10%	37%	46%	-3.0%	-1.9%	-0.7%	-0.9%		5%	9%	32%	41%	-3.0%	-1.4%	-0.6%	-0.8%
**Mauritius**	2%	5%	18%	23%	0.3%	0.7%	-0.8%	-0.5%		2%	10%	31%	39%	0.7%	0.2%	-0.9%	-0.5%		2%	7%	25%	31%	0.5%	0.4%	-0.8%	-0.5%
**Mexico**	2%	4%	18%	23%	-3.1%	0.1%	-1.0%	-0.9%		2%	12%	26%	36%	-3.0%	0.7%	-0.3%	-0.1%		2%	8%	22%	29%	-3.1%	0.6%	-0.6%	-0.4%

**Mongolia**	2%	5%	21%	26%	-6.1%	-3.6%	-3.5%	-3.4%		2%	14%	42%	51%	-6.3%	-2.4%	-1.1%	-1.3%		2%	10%	31%	39%	-6.2%	-2.7%	-2.1%	-2.1%
**Montenegro**	1%	3%	14%	17%	-5.2%	-1.8%	-1.3%	-1.5%		1%	6%	28%	33%	-5.3%	0.5%	-0.4%	-0.4%		1%	4%	21%	25%	-5.2%	-0.3%	-0.7%	-0.7%
**Morocco**	2%	4%	17%	22%	-5.3%	-4.7%	-3.3%	-3.4%		2%	5%	27%	33%	-5.2%	-4.0%	-1.6%	-2.0%		2%	4%	22%	28%	-5.2%	-4.3%	-2.2%	-2.5%
**Mozambique**	8%	13%	34%	47%	-4.2%	-6.6%	-2.7%	-3.0%		9%	20%	46%	61%	-3.9%	-3.1%	-1.1%	-1.4%		8%	16%	39%	53%	-4.1%	-4.6%	-2.0%	-2.2%
**Myanmar**	5%	8%	27%	36%	-4.0%	-2.8%	-1.1%	-1.5%		6%	14%	39%	51%	-3.5%	-1.9%	-0.7%	-1.0%		5%	11%	33%	44%	-3.7%	-2.2%	-0.9%	-1.2%
**Namibia**	5%	14%	33%	45%	-2.5%	-7.9%	-3.4%	-3.7%		5%	23%	48%	62%	-2.6%	-5.0%	-1.9%	-2.0%		5%	19%	40%	54%	-2.5%	-6.1%	-2.6%	-2.8%
**Nepal**	4%	8%	26%	34%	-4.6%	-2.0%	-0.8%	-1.2%		4%	9%	34%	42%	-4.3%	-2.3%	-0.8%	-1.1%		4%	8%	30%	38%	-4.5%	-2.2%	-0.8%	-1.2%
**Netherlands**	0%	2%	10%	12%	-1.3%	-1.7%	-1.1%	-1.2%		1%	3%	14%	16%	-1.2%	-1.3%	-2.2%	-2.0%		0%	2%	12%	14%	-1.2%	-1.4%	-1.8%	-1.7%
**New Zealand**	1%	2%	9%	12%	0.1%	-1.3%	-1.0%	-1.0%		1%	4%	14%	18%	-1.1%	-0.7%	-0.7%	-0.7%		1%	3%	11%	15%	-0.6%	-0.9%	-0.9%	-0.8%
**Nicaragua**	2%	4%	19%	24%	-3.2%	-2.6%	-1.1%	-1.4%		2%	9%	27%	35%	-3.8%	-2.4%	-1.2%	-1.4%		2%	7%	23%	30%	-3.6%	-2.5%	-1.2%	-1.4%
**Niger**	13%	12%	29%	46%	-1.8%	-2.4%	-1.3%	-1.3%		14%	13%	36%	52%	-1.6%	-1.8%	-0.8%	-0.9%		14%	12%	32%	49%	-1.7%	-2.1%	-1.0%	-1.1%
**Nigeria**	17%	24%	40%	62%	-1.2%	-0.3%	-0.2%	-0.3%		17%	24%	43%	64%	-1.5%	-0.5%	-0.3%	-0.4%		17%	24%	42%	63%	-1.4%	-0.4%	-0.2%	-0.3%
**North Macedonia**	1%	2%	15%	18%	-2.8%	-2.5%	-1.3%	-1.4%		1%	4%	26%	29%	-1.9%	-2.7%	-1.5%	-1.6%		1%	3%	20%	23%	-2.3%	-2.6%	-1.4%	-1.5%
**Norway**	0%	1%	8%	10%	-3.3%	-3.2%	-2.6%	-2.7%		0%	3%	12%	15%	-2.0%	-2.7%	-2.9%	-2.8%		0%	2%	10%	12%	-2.6%	-2.8%	-2.8%	-2.7%
**Oman**	1%	2%	10%	12%	-0.8%	-3.9%	-3.9%	-3.6%		1%	3%	14%	18%	-1.5%	-5.1%	-3.8%	-3.7%		1%	2%	12%	16%	-1.2%	-4.6%	-3.8%	-3.6%
**Pakistan**	7%	7%	25%	34%	-3.1%	-1.8%	-0.8%	-1.2%		8%	10%	35%	46%	-2.6%	-1.3%	-0.4%	-0.7%		7%	8%	30%	41%	-2.8%	-1.5%	-0.6%	-0.9%
**Panama**	2%	3%	11%	16%	-0.8%	-1.6%	-1.2%	-1.2%		2%	7%	18%	26%	-0.9%	-2.9%	-1.7%	-1.8%		2%	5%	15%	21%	-0.8%	-2.5%	-1.5%	-1.6%
**Papua New Guinea**	5%	9%	29%	38%	-3.1%	-2.8%	-1.2%	-1.5%		6%	14%	43%	54%	-2.6%	-1.0%	-0.3%	-0.5%		5%	12%	37%	47%	-2.8%	-1.8%	-0.6%	-0.9%
**Paraguay**	2%	4%	18%	23%	-2.9%	-2.1%	-0.9%	-1.2%		2%	9%	29%	37%	-2.9%	0.3%	-0.3%	-0.3%		2%	7%	24%	30%	-2.9%	-0.5%	-0.6%	-0.7%
**Peru**	2%	5%	16%	22%	-3.1%	-2.7%	-3.1%	-2.8%		2%	8%	21%	29%	-3.3%	-2.9%	-2.9%	-2.7%		2%	6%	19%	25%	-3.2%	-2.9%	-3.0%	-2.8%
**Philippines**	3%	6%	23%	30%	-0.7%	-0.6%	-0.5%	-0.5%		4%	11%	38%	47%	-0.7%	-1.0%	-0.8%	-0.8%		3%	9%	30%	39%	-0.7%	-0.9%	-0.5%	-0.5%
**Poland**	1%	2%	13%	16%	-2.5%	-1.9%	-0.9%	-1.1%		1%	7%	29%	34%	-3.6%	-2.1%	-1.2%	-1.3%		1%	4%	21%	25%	-3.1%	-2.0%	-1.0%	-1.1%
**Portugal**	0%	2%	8%	10%	-0.8%	-2.8%	-1.3%	-1.5%		0%	4%	18%	22%	0.3%	-4.4%	-1.0%	-1.5%		0%	3%	13%	16%	-0.2%	-4.0%	-1.1%	-1.5%
**Puerto Rico**	1%	2%	9%	11%	-1.9%	-5.6%	-2.6%	-2.9%		1%	6%	21%	26%	-3.1%	-3.6%	-0.9%	-1.4%		1%	4%	15%	18%	-2.6%	-4.1%	-1.6%	-2.0%
**Qatar**	1%	1%	9%	10%	-4.2%	-4.7%	-5.9%	-5.6%		1%	2%	8%	10%	-4.3%	-5.8%	-5.0%	-5.0%		1%	2%	8%	10%	-4.3%	-5.8%	-5.4%	-5.2%
**Republic of Korea**	0%	1%	6%	7%	-3.8%	-5.1%	-4.1%	-4.1%		0%	2%	15%	17%	-3.7%	-6.3%	-3.6%	-3.8%		0%	1%	10%	12%	-3.7%	-6.0%	-3.8%	-3.9%
**Republic of Moldova**	2%	5%	23%	29%	-1.3%	0.5%	-1.1%	-0.9%		2%	14%	45%	54%	-1.6%	0.2%	0.1%	0.0%		2%	9%	33%	41%	-1.5%	0.3%	-0.4%	-0.3%
**Romania**	1%	3%	16%	19%	-5.9%	-2.8%	-1.0%	-1.4%		1%	7%	35%	40%	-6.3%	-2.7%	-0.2%	-0.7%		1%	5%	25%	29%	-6.1%	-2.7%	-0.5%	-0.9%
**Russian Federation**	1%	5%	17%	22%	-8.2%	-3.5%	-2.6%	-2.8%		1%	15%	41%	50%	-7.6%	-4.6%	-2.3%	-2.4%		1%	10%	28%	36%	-7.9%	-4.3%	-2.4%	-2.7%

**Rwanda**	5%	10%	29%	39%	-4.9%	-3.7%	-1.6%	-2.0%		5%	14%	39%	50%	-4.8%	-2.1%	-0.5%	-1.0%		5%	12%	34%	44%	-4.9%	-2.8%	-1.0%	-1.4%
**Saudi Arabia**	1%	2%	14%	16%	-5.0%	-3.6%	-2.5%	-2.7%		1%	4%	17%	22%	-4.9%	-3.2%	-4.4%	-4.0%		1%	4%	16%	20%	-4.9%	-3.6%	-3.8%	-3.6%
**Senegal**	5%	8%	24%	34%	-5.0%	-2.5%	-1.1%	-1.7%		6%	11%	33%	44%	-4.6%	-2.3%	-1.0%	-1.4%		5%	10%	29%	39%	-4.8%	-2.4%	-1.0%	-1.5%
**Serbia**	1%	3%	16%	19%	-3.5%	-2.2%	-1.2%	-1.4%		1%	5%	31%	35%	-3.4%	-2.3%	-0.8%	-0.9%		1%	4%	23%	27%	-3.4%	-2.3%	-0.9%	-1.1%
**Seychelles**	2%	5%	19%	24%	1.2%	0.6%	0.2%	0.3%		2%	10%	35%	43%	1.1%	0.1%	-0.2%	-0.1%		2%	8%	28%	35%	1.1%	0.5%	0.0%	0.1%
**Sierra Leone**	12%	13%	34%	49%	-4.6%	-5.5%	-2.0%	-2.5%		14%	14%	40%	55%	-4.0%	-5.1%	-1.7%	-2.0%		13%	13%	37%	52%	-4.3%	-5.3%	-1.9%	-2.3%
**Singapore**	0%	1%	7%	9%	-2.0%	-2.8%	-3.0%	-2.9%		0%	2%	13%	15%	-2.4%	-2.8%	-2.8%	-2.7%		0%	2%	10%	12%	-2.3%	-2.9%	-2.8%	-2.8%
**Slovakia**	1%	2%	13%	15%	-1.8%	-0.9%	-1.5%	-1.4%		1%	5%	28%	32%	-1.6%	-2.6%	-1.9%	-1.8%		1%	4%	20%	24%	-1.6%	-2.1%	-1.7%	-1.7%
**Slovenia**	0%	1%	9%	11%	-5.0%	-3.7%	-1.1%	-1.5%		0%	3%	19%	22%	-0.8%	-3.3%	-1.9%	-2.0%		0%	2%	14%	17%	-2.6%	-3.4%	-1.6%	-1.8%
**Solomon Islands**	2%	8%	26%	34%	-2.5%	-0.4%	-0.2%	-0.3%		3%	9%	34%	42%	-3.0%	-2.0%	-0.7%	-0.9%		3%	8%	30%	38%	-2.8%	-1.3%	-0.5%	-0.7%
**Somalia**	14%	16%	32%	51%	-5.3%	-3.3%	-2.2%	-2.4%		15%	21%	38%	59%	-4.8%	-2.9%	-2.0%	-1.9%		15%	18%	35%	55%	-5.0%	-3.1%	-2.1%	-2.1%
**South Africa**	4%	14%	29%	41%	-4.9%	-7.5%	-1.4%	-3.0%		5%	20%	44%	57%	-4.5%	-5.5%	-1.3%	-2.0%		4%	17%	36%	49%	-4.7%	-6.3%	-1.4%	-2.4%
**South Sudan**	12%	16%	32%	49%	-1.4%	-1.0%	-0.5%	-0.6%		14%	22%	40%	59%	-0.7%	1.3%	0.5%	0.4%		13%	19%	35%	54%	-1.0%	0.3%	0.0%	-0.1%
**Spain**	0%	1%	7%	9%	-2.8%	-2.6%	-0.2%	-0.6%		0%	2%	15%	18%	-2.0%	-3.8%	-1.4%	-1.6%		0%	2%	11%	13%	-2.3%	-3.4%	-1.0%	-1.4%
**Sri Lanka**	1%	3%	13%	16%	-4.6%	-2.7%	-1.6%	-1.8%		1%	6%	28%	33%	-4.6%	-3.8%	-1.4%	-1.7%		1%	4%	20%	24%	-4.6%	-3.5%	-1.5%	-1.8%
**State of Palestine**	2%	3%	15%	19%	-3.6%	-3.5%	-3.0%	-3.0%		2%	4%	25%	29%	-3.5%	-2.8%	-1.4%	-1.6%		2%	4%	20%	24%	-3.5%	-3.2%	-2.1%	-2.2%
**Sudan**	6%	9%	24%	36%	-3.2%	-2.1%	-1.1%	-1.4%		8%	15%	33%	47%	-2.6%	-1.4%	-0.7%	-0.9%		7%	12%	29%	42%	-2.9%	-1.7%	-1.0%	-1.2%
**Suriname**	2%	7%	22%	29%	-1.9%	-1.5%	-0.7%	-0.9%		2%	10%	36%	44%	-2.6%	-2.9%	-0.5%	-0.9%		2%	9%	29%	36%	-2.3%	-2.5%	-0.5%	-0.9%
**Sweden**	0%	2%	8%	10%	-2.5%	-0.5%	-2.0%	-1.8%		0%	3%	12%	15%	-2.7%	-1.5%	-2.2%	-2.0%		0%	2%	10%	12%	-2.6%	-1.1%	-2.1%	-1.9%
**Switzerland**	0%	1%	7%	9%	-2.4%	-3.0%	-1.7%	-1.9%		0%	2%	12%	14%	-0.7%	-3.0%	-2.3%	-2.3%		0%	2%	10%	12%	-1.6%	-3.0%	-2.0%	-2.1%
**Syrian Arab Republic**	3%	5%	20%	26%	3.5%	2.1%	0.8%	1.2%		3%	12%	32%	42%	3.0%	8.1%	1.1%	2.1%		3%	8%	26%	34%	3.2%	6.0%	0.8%	1.7%
**Tajikistan**	3%	5%	23%	29%	-2.9%	-2.4%	-1.5%	-1.6%		4%	7%	33%	41%	-2.8%	-3.9%	-1.5%	-1.7%		4%	6%	28%	35%	-2.9%	-3.2%	-1.6%	-1.7%
**Thailand**	1%	4%	12%	17%	-3.5%	-1.3%	-1.8%	-1.7%		1%	12%	25%	35%	-3.3%	-0.3%	-0.1%	-0.3%		1%	8%	19%	26%	-3.4%	-0.6%	-0.7%	-0.8%
**Timor-Leste**	6%	9%	28%	38%	-3.6%	-2.5%	-1.0%	-1.4%		7%	11%	36%	47%	-3.1%	-2.1%	-0.7%	-1.0%		6%	10%	32%	43%	-3.3%	-2.3%	-0.8%	-1.1%
**Togo**	9%	17%	35%	50%	-3.1%	-1.9%	-1.1%	-1.2%		9%	16%	38%	53%	-3.2%	-2.4%	-1.1%	-1.3%		9%	16%	36%	51%	-3.2%	-2.2%	-1.1%	-1.3%
**Tonga**	1%	5%	20%	25%	-1.4%	-1.0%	-0.6%	-0.7%		2%	11%	36%	44%	-1.5%	-0.9%	-0.6%	-0.6%		1%	8%	28%	35%	-1.5%	-1.1%	-0.6%	-0.7%
**Trinidad and Tobago**	2%	5%	18%	23%	-2.4%	-0.4%	-1.3%	-1.1%		2%	11%	28%	38%	-2.2%	0.1%	-1.0%	-0.7%		2%	8%	23%	31%	-2.3%	0.0%	-1.1%	-0.9%
**Tunisia**	2%	3%	14%	18%	-3.0%	-1.6%	-1.2%	-1.4%		2%	6%	25%	31%	-3.0%	-0.3%	-0.6%	-0.7%		2%	4%	20%	24%	-3.1%	-0.7%	-0.8%	-0.9%
**Turkmenistan**	4%	5%	23%	30%	-0.2%	-1.3%	-0.2%	-0.4%		5%	10%	38%	47%	-0.3%	-1.7%	-0.2%	-0.4%		5%	7%	29%	38%	-0.2%	-1.6%	-0.3%	-0.4%

**Türkiye**	1%	2%	12%	14%	-5.7%	-3.7%	-2.6%	-2.9%		1%	4%	25%	29%	-5.6%	-3.8%	-1.8%	-2.0%		1%	3%	18%	22%	-5.6%	-3.8%	-2.0%	-2.3%
**Uganda**	5%	12%	31%	42%	-5.8%	-5.4%	-2.4%	-2.8%		6%	16%	41%	54%	-6.0%	-3.6%	-1.1%	-1.6%		5%	14%	36%	48%	-5.9%	-4.4%	-1.7%	-2.2%
**Ukraine**	1%	4%	16%	20%	-3.8%	-3.0%	-2.9%	-2.8%		1%	13%	38%	47%	-4.7%	-2.9%	-1.8%	-1.8%		1%	9%	26%	33%	-4.3%	-3.0%	-2.0%	-2.1%
**United Arab Emirates**	1%	1%	6%	8%	-2.7%	-3.5%	-1.3%	-1.6%		1%	2%	8%	11%	-3.3%	-2.1%	0.3%	-0.5%		1%	2%	8%	10%	-3.0%	-2.6%	-0.3%	-0.9%
**United Kingdom**	0%	2%	10%	13%	-2.0%	-0.2%	-1.0%	-0.9%		1%	4%	15%	19%	-1.6%	-0.4%	-1.2%	-1.0%		1%	3%	13%	16%	-1.8%	-0.3%	-1.1%	-1.0%
**United Republic of Tanzania**	5%	11%	30%	41%	-4.7%	-5.2%	-2.3%	-2.7%		6%	16%	41%	53%	-4.7%	-2.4%	-0.7%	-1.1%		5%	14%	35%	47%	-4.7%	-3.6%	-1.5%	-1.9%
**United States of America**	1%	4%	13%	17%	-1.0%	0.7%	-0.3%	-0.1%		1%	7%	21%	27%	-1.0%	1.2%	0.0%	0.2%		1%	5%	17%	22%	-1.0%	1.0%	-0.1%	0.1%
**Uruguay**	1%	4%	14%	18%	-2.5%	0.0%	0.0%	-0.1%		1%	7%	26%	32%	-3.0%	0.3%	-1.1%	-0.8%		1%	6%	20%	25%	-2.8%	0.2%	-0.7%	-0.5%
**Uzbekistan**	2%	5%	21%	26%	-6.2%	-1.3%	-1.7%	-1.8%		2%	9%	36%	43%	-6.0%	-0.8%	-0.4%	-0.6%		2%	7%	28%	35%	-6.1%	-1.0%	-0.9%	-1.1%
**Vanuatu**	2%	7%	23%	30%	-2.0%	-0.4%	-0.4%	-0.5%		3%	9%	35%	42%	-2.1%	-1.2%	-0.7%	-0.7%		2%	8%	29%	37%	-2.1%	-0.9%	-0.8%	-0.8%
**Venezuela**	2%	5%	17%	23%	0.9%	1.9%	0.7%	0.9%		2%	13%	27%	39%	0.6%	-0.2%	0.3%	0.2%		2%	10%	22%	31%	0.8%	0.3%	0.4%	0.4%
**Viet Nam**	2%	4%	13%	18%	-0.9%	-0.5%	-0.5%	-0.5%		4%	11%	28%	37%	-1.1%	-0.5%	-0.3%	-0.3%		3%	7%	20%	28%	-1.0%	-0.4%	-0.3%	-0.4%
**Yemen**	5%	7%	25%	34%	-1.9%	-0.3%	-0.3%	-0.4%		6%	16%	37%	50%	-1.7%	5.1%	0.5%	1.0%		5%	12%	31%	42%	-1.8%	3.3%	0.2%	0.5%
**Zambia**	7%	12%	31%	44%	-3.3%	-7.4%	-3.3%	-3.5%		8%	18%	42%	56%	-3.0%	-3.8%	-1.7%	-1.8%		7%	15%	36%	50%	-3.1%	-5.4%	-2.4%	-2.5%
**Zimbabwe**	6%	19%	38%	53%	-5.2%	-7.3%	-3.2%	-3.4%		7%	23%	47%	62%	-5.2%	-4.9%	-1.7%	-2.0%		6%	21%	42%	57%	-5.2%	-6.2%	-2.5%	-2.7%

Table A4. Years lived with disability (YLD) and proportion of YLD over life expectancy. Calculated from healthy life expectancy (HALE) and life expectancy at birth in 2019 for the 30 most populous countries. Both sexes combined. *Source: WHO Global Health Observatory*[4]

	Healthy life years	Life expectancy at birth	Years lived with disability	Proportion YLD/LE
Japan	74.1	84.3	10.2	12%
Republic of Korea	73.1	83.3	10.2	12%
Spain	72.1	83.2	11.1	13%
Italy	71.9	83.0	11.1	13%
France	72.1	82.5	10.4	13%
Germany	70.9	81.7	10.8	13%
UK	70.1	81.4	11.3	14%
Colombia	69.0	79.3	10.3	13%
Türkiye	68.4	78.6	10.2	13%
USA	66.1	78.5	12.4	16%
Thailand	68.3	77.7	9.4	12%
China	68.5	77.4	8.9	11%
Iran	66.3	77.3	11	14%
Mexico	65.8	76	10.2	13%
Brazil	65.4	75.9	10.5	14%
Bangladesh	64.3	74.3	10	13%
Viet Nam	65.3	73.7	8.4	11%
Russia	64.2	73.2	9	12%
Egypt	63.0	71.8	8.8	12%
Indonesia	62.8	71.3	8.5	12%
India	60.3	70.8	10.5	15%
Philippines	62.0	70.4	8.4	12%
Myanmar	60.9	69.1	8.2	12%
Ethiopia	59.9	68.7	8.8	13%
United Republic of Tanzania	58.5	67.3	8.8	13%
Kenya	57.7	66.1	8.4	13%
Pakistan	56.9	65.6	8.7	13%
South Africa	56.2	65.3	9.1	14%
Nigeria	54.4	62.6	8.2	13%

References

1. Aburto JM, Villavicencio F, Basellini U, Kjaergaard S, Vaupel JW. Dynamics of life expectancy and life span equality. *Proc Natl Acad Sci U S A* 2020; **117**(10): 5250-9.
2. Salomon JA, Wang H, Freeman MK, et al. Healthy life expectancy for 187 countries, 1990-2010: a systematic analysis for the Global Burden Disease Study 2010. *Lancet* 2012; **380**(9859): 2144-62.
3. Mathers CD, Sadana R, Salomon JA, Murray CJ, Lopez AD. Healthy life expectancy in 191 countries, 1999. *Lancet* 2001; **357**(9269): 1685-91.
4. WHO Global Health Observatory. Healthy life expectancy (HALE) at birth. 2021. https://www.who.int/data/gho/data/indicators/indicator-details/GHO/gho-ghe-hale-healthy-life-expectancy-at-birth (accessed May 2, 2024).
5. Diseases GBD, Injuries C. Global incidence, prevalence, years lived with disability (YLDs), disability-adjusted life-years (DALYs), and healthy life expectancy (HALE) for 371 diseases and injuries in 204 countries and territories and 811 subnational locations, 1990-2021: a systematic analysis for the Global Burden of Disease Study 2021. *Lancet* 2024; **403**(10440): 2133-61.
6. United Nations Population Division. World Population Prospects. 2024. https://population.un.org/wpp/Download/Standard/Population/ (accessed July 11, 2024).
